# Current transition management of adolescents and young adults with allergy and asthma: a European survey

**DOI:** 10.1186/s13601-020-00340-z

**Published:** 2020-10-07

**Authors:** Ekaterina Khaleva, Marta Vazquez-Ortiz, Pasquale Comberiati, Audrey DunnGalvin, Helena Pite, Katharina Blumchen, Teresa Garriga-Baraut, Valerie Hox, Alexandra F. Santos, Claudia Gore, Rebecca C. Knibb, Cherry Alviani, Charlotte G. Mortz, Elizabeth Angier, Bettina Duca, Britt Jensen, Silvia Sanchez-Garcia, M. Hazel Gowland, Frans Timmermans, Oliver Pfaar, Graham Roberts

**Affiliations:** 1 Faculty of Medicine, University of Southampton, Southampton, UK; 2 Section of Inflammation, Repair and Development, National Heart and Lung Institute, Imperial College London, London, UK; 3 Department of Clinical and Experimental Medicine, Section of Paediatrics, University of Pisa, Pisa, Italy; 4 Department of Clinical Immunology and Allergology, I.M. Sechenov First Moscow State Medical University, Moscow, Russia; 5 Applied Psychology and Paediatrics and Child Health, University College Cork, Cork, Ireland; 6 Paediatrics and Child Infectious Diseases, First Moscow State Medical University, Moscow, Russia; 7 Allergy Center, CUF Descobertas Hospital and CUF Infante Santo Hospital, Lisbon, Portugal; 8 CEDOC, Chronic Diseases Research Center, NOVA Medical School/Faculdade de Ciências Médicas, Universidade Nova de Lisboa, Lisbon, Portugal; 9 Department of Paediatric and Adolescent Medicine, Paediatric Pneumology, Allergology and Cystic Fibrosis, University Hospital Frankfurt, Frankfurt Am Main, Germany; 10 Unitat d’Allergologia Pediàtrica, Hospital Universitari Vall d’Hebron, Barcelona, Spain; 11 Grup d’Investigació “Creixement i Desenvolupament”, Institut de Recerca de l’Hospital Universitari Vall d’Hebron (VHIR), Barcelona, Spain; 12 Department of Otorhinolaryngology, Head and Neck Surgery, University Hospitals Saint-Luc, Brussels, Belgium; 13 Department of Women and Children’s Health (Paediatric Allergy, School of Life Course Sciences, Faculty of Life Sciences and Medicine, King’s College London, London, UK; 14 Peter Gorer Department of Immunobiology, School of Immunology and Microbial Sciences, King’s College London, London, UK; 15 Children’s Allergy Service, Guy’s and St Thomas’ Hospital, London, UK; 16 Asthma UK Centre in Allergic Mechanisms of Asthma, London, UK; 17 Department of Paediatrics, Imperial College Healthcare NHS Trust, London, UK; 18 Department of Psychology, School of Life and Health Sciences, Aston University, Birmingham, UK; 19 The David Hide Asthma and Allergy Research Centre, St Mary’s Hospital, Isle of Wight, UK; 20 Department of Dermatology and Allergy Centre, Odense Research Centre for Anaphylaxis (ORCA), Odense University Hospital, Odense, Denmark; 21 Primary Care and Population Sciences, University of Southampton, Southampton, UK; 22 Allergy Department, Hospital Infantil Universitario del Niño Jesús, Madrid, Spain; 23 Allergy Action, St Albans, UK; 24 Nederlands Anafylaxis Netwerk – European Anaphylaxis Taskforce, Dordrecht, The Netherlands; 25 Department of Otorhinolaryngology, Head and Neck Surgery, Section of Rhinology and Allergy, University Hospital Marburg, Philipps-Universität Marburg, Marburg, Germany; 26 NIHR Southampton Biomedical Research Centre, University Hospital Southampton NHS Foundation Trust, Southampton, UK

**Keywords:** Adolescent, Allergy, Healthcare professional, Transition, Young adult

## Abstract

**Background:**

Transition from parent-delivered to self-management is a vulnerable time for adolescents and young adults (AYA) with allergy and asthma. There is currently no European guideline available for healthcare professionals (HCPs) on transition of these patients and local/national protocols are also mostly lacking.

**Methods:**

European HCPs working with AYA with allergy and asthma were invited to complete an online survey assessing challenges of working with these patients, current transition practices and access to specific healthcare resources.

**Results:**

A total of 1179 responses from 41 European countries were collected. Most HCPs (86%) reported a lack of a transition guideline and a lack of a transition process (20% paediatric HCPs, 50% of adult HCPs, 56% HCP seeing all ages). Nearly half (48%) acknowledged a lack of an established feedback system between paediatric and adult medical services. Many respondents never routinely asked about mental health issues such as self-harm or depression and are not confident in asking about self-harm (66.6%), sexuality (64%) and depression (43.6%). The majority of HCPs (76%) had not received specific training in the care of AYA although 87% agreed that transition was important for AYA with allergy and asthma.

**Conclusion:**

Although there was agreement that transition is important for AYA with allergy and asthma, there are crucial limitations and variations in the current provision of transition services across Europe. Standardisation of AYA management and specific training are required. This should improve management and continuity of care during adolescence and into adulthood to achieve the best healthcare outcomes.
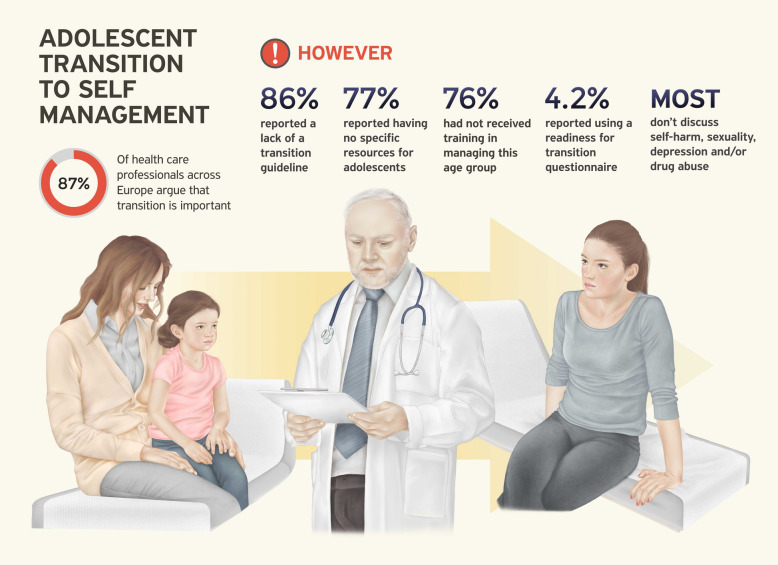

## Introduction

Allergy and asthma are amongst the most common chronic disorders. Furthermore, the prevalence and severity of allergic diseases and asthma continue to rise with adolescents and young adults (AYA)—those between ages 11 and 25 [1–3]. It has been shown that AYA have higher rates of fatal anaphylaxis to foods [4] and asthma deaths compared to younger children [5] partly due to risk taking behaviour and poor adherence. Moreover, AYA with food allergy (FA) have a lower quality of life (QoL) than AYA with other chronic conditions [6]. These findings suggest that AYA with allergic conditions require specialised resources and healthcare plans to address their age- and disease-related needs.

Adolescence and young adulthood is an important period of development with significant biological, psychological and social changes [7]. As adolescents move towards adulthood, there is a need to evolve from being dependent on their parents/carers to becoming responsible and accountable for their own health and well-being as adults. This is independent of whether there are separate paediatric and adult allergy clinics or one allergy clinic seeing all age groups. Transition has been defined as ‘active and evolving process that addresses the medical, psychosocial, and educational needs of young people as they prepare to move from child- to adult-centred health care’ [8]. So it is not only about transfer of patient information and disease history to an adult healthcare setting. Transition also, importantly, includes the provision of the support that AYA with long-term allergic conditions require to meet their needs to progress to being independent adult patients.

Previous studies have shown that AYA and their parents are mostly dissatisfied with their experience of the transition process. For instance, only 42% of AYA with special healthcare needs had discussed transition care with their healthcare professional (HCP) and only 41% met the transition core outcomes such as whether HCP had discussed transition to adult medical service, health care needs, health insurance and had encouraged the AYA to self-manage their disease [9]. AYA with sickle cell disease have voiced concern about the care they will receive in adult healthcare services, being worried about leaving a familiar and trusted paediatric doctor [10]. A recent systematic review on the challenges faced by AYA with allergy and asthma identified a large number of fixed and modifiable factors, including psychological, social/environmental, behavioural factors as well as the nature of the patient-HCP relationship, that will influence self-management and ultimately health outcomes [11]. In addition, a related systematic review assessed different interventions for improving self-management and wellbeing of AYA with asthma and allergies; many delivered improvements in patients with asthma although more robust evidence is required, especially for other allergic diseases [12]. Given this complexity we need to find ways for HCPs to facilitate the smooth transition process from a paediatric to adult format of medical care and inform transition guidelines.

During the last decade, a number of transition models and guidelines have been proposed to address the organization and process of transition. There are no conclusive data on the superiority of one transition programme over another [13]. The need for a multidisciplinary service model integrating social support, education and non-statutory services is well established [14] and exemplified by the recently published European Academy of Paediatrics consensus statement [15]. Furthermore, a number of disease-specific programmes have been set up to address the process of the transition such as in patients with chronic digestive [16], rheumatic [17, 18], liver [8] and coeliac [19] diseases.

To our knowledge, there are currently no standardized policies and protocols on the transition of AYA with allergy and asthma in most European countries. Moreover, there are currently no international or European accepted guidelines available for HCPs working in this field. To develop best transition practices for AYA with allergic diseases across Europe, it is first necessary to understand current transition care, as well as the barriers and facilitators HCPs face to implement quality of care. This paper describes the results of a pan-European survey to assess the challenges of working with AYA, current transition practices and access to specific healthcare resources to support transition.

## Methods

### Study design

A quantitative, online, cross-sectional survey was conducted. As no relevant validated questionnaire existed, the survey was developed by the members of the European Academy of Allergy and Clinical Immunology (EAACI) Adolescent and Young Adult Task Force after a systematic literature review on the transition process and challenges of the AYA with allergies. The study was approved by the Ethics and Research Governance Committee at University of Southampton, United Kingdom.

### Participants

We invited HCPs managing AYA with allergy and asthma across Europe and members of the EAACI and/or National Allergy Societies (NAS) within Europe who were able to read English, German, French, Greek, Spanish, Portuguese, Italian or Russian to participate in the survey. The potential survey population was approximately 12,000 participants, the number of EAACI members, in addition to members of the NAS. As it was not possible to identify non-clinicians, the survey was sent to all EAACI members and it was highlighted in the invitation email that the survey was only for HCPs. Participants were asked to fill in the survey only once. A margin of error for answers to questions was set at 5% with a confidence level of 95%. For this, the SurveyMonkey tool (https://www.surveymonkey.com/mp/sample-size-calculator/) indicated that a sample size of 373 participants was required to provide good estimates given the overall population size of 12,000.

### Data collection

The survey was distributed by the scientific content officer of EAACI and presidents of NAS in Europe via a link to the survey in SurveyMonkey through the members’ mailing list. In addition, the survey was advertised on social media (e.g. Facebook, Twitter) and during the EAACI 2019 congress. Before accessing the questionnaire, potential respondents were informed about the study’s purpose, average time required to complete the survey and confidentiality policy on the last page of the SurveyMonkey. The survey was conducted between 30th May and 28th June 2019. Two reminder emails were sent.

### The questionnaire

The anonymized survey consisted of 25 questions (see supplementary materials). The questionnaire was translated into eight languages (English, German, French, Greek, Spanish, Portuguese, Italian and Russian) and back-translated into English to ensure validity. To reduce measurement error, some words, which could have several meanings or did not have a direct translation such as transition, transition lead, transition readiness assessment tool and transition report were described in the glossary at the beginning of the survey. A pilot on-line survey was conducted with 20 volunteer HCPs from the target group in different countries who were not members of the EAACI Task Force to optimize clarity, relevance and web administration. They also tested the time required to complete the survey, which ranged from 8 to 11 min.

To enhance completion rates for the survey and to keep it brief, minimal demographic and training information was collected. An option for other free-text response was permitted in each question. Data from the free-text answers was coded as ‘other’ and described in the footnotes of tables and figures.

### Statistical analysis

All data was collected and analyzed using SPSS software version 25.0. Descriptive statistics were used to describe respondent characteristics. Means, medians, standard deviations, and lower and upper quartiles are presented for continuous variables. Frequency tables with percentages are provided for categorical variables. Categorical variables were compared using Chi square or Fisher’s exact test as appropriate. Association between clinic type (paediatric, all ages groups HCP), countries with more than 50 responses and investigated parameters were assessed by multiple regression analysis.

Two sub-analyses were performed, one amongst HCPs from different geographic regions and one amongst pediatricians, adults, or all ages HCPs. A minimum of 50 responders per country was required for comparison of data between countries to ensure that there was adequate power to detect significant differences. Summary tables and bar charts were used to represent the results. Data was considered significant if statistical tests produce a *p* value of < 0.05.

A qualitative data analysis was used to summarise HCP’s comments. Text was divided into separate units, coded and summarized as themes. Each response was reviewed by EK and GR. Any discrepancies were resolved through discussion and, if necessary, a third reviewer (MVO) was consulted.

## Results

### Respondent demographics and characteristics

We received 1819 responses, 550 were incomplete and 14 were excluded as they did not satisfy the inclusion criteria. A total of 1255 responses from 71 countries were analysed. Further analysis focused on the 1179 responses which came from Europe. There were 449 (38.1%) paediatricians, 88 (7.5%) adult physicians and 642 (54.5%) HCPs who see all age groups. Respondent’s’ characteristics are listed in Table 1. Additionally, a sensitivity analysis was performed looking at difference between responses in different languages. These findings were similar to those for the comparison between different countries (Additional file 1: Tables S1, S2).

**Table 1:** Demographics of survey responders and practice characteristics

European countries (n = 1179)	Number (%) respondents
Albania	2 (0.2)
Austria	6 (0.5)
Belarus	8 (0.6)
Belgium	7 (0.6)
Bulgaria	4 (0.3)
Croatia	3 (0.2)
Cyprus	1 (0.1)
Czech Republic	26 (2.1)
Denmark	30 (2.4)
Estonia	4 (0.3)
Finland	4 (0.3)
France	46 (3.7)
Germany	68 (5.4)
Greece	34 (2.7)
Hungary	3 (0.2)
Iceland	3 (0.2)
Ireland	31 (2.5)
Italy	110 (8.8)
Kazakhstan	1 (0.1)
Kosovo	2 (0.2)
Latvia	1 (0.1)
Lithuania	6 (0.5)
Moldova	1 (0.1)
Monaco	1 (0.1)
Netherlands	32 (2.5)
Norway	16 (1.3)
Poland	10 (0.8)
Portugal	56 (4.5)
Republic of North Macedonia	8 (0.6)
Romania	54 (4.3)
Russia	175 (13.9)
Serbia	10 (0.8)
Slovakia	19 (1.5)
Slovenia	10 (0.8)
Spain	170 (13.5)
Sweden	29 (2.3)
Switzerland	8 (0.6)
Turkey	35 (2.8)
Ukraine	19 (1.5)
United Kingdom	124 (9.9)
Uzbekistan	2 (0.2)
Non-European countries^a^	76 (6.1)
Language	
English	537 (45.5)
Italian	105 (8.9)
Greek	26 (2.2)
Spanish	146 (12.4)
German	74 (6.3)
Russian	204 (17.3)
French	34 (2.9)
EAACI section	
Asthma	292 (24.8)
Dermatology	57 (4.8)
ENT	46 (3.9)
Immunology	99 (8.4)
Paediatrics	358 (30.4)
Primary Care and Allied Health	51 (4.3)
None^b^	276 (23.4)
Profession	
Doctor	1082 (91.8)
Specialist allergy nurse	68 (5.8)
Dietician	15 (1.3)
Others^c^	14 (1.2)
Speciality^e^	
Paediatric allergy	368 (31.2)
Paediatrics	331 (28.1)
Allergy (adults only)	138 (11.7)
Allergy (children and adults)	514 (43.6)
Dermatology	40 (3.4)
Respiratory Medicine	172 (14.6)
Otorhinolaryngology	37 (3.1)
General practitioner	41 (3.5)
Internal Medicine	11 (0.9)
Immunology	16 (1.4)
Others^d^	35 (3.0)
Work setting^e^	
Tertiary care	542 (46)
Secondary care	293 (24.9)
Primary care	270 (22.9)
Private practice	283 (24.0)
Research	7 (0.6)
Years in practice	
0–5	248 (21)
6–10	261 (22.1)
11–20	371 (31.5)
> 21	299 (25.3)

*ENT* otolaryngology

^a^Non-European countries (Supplementary materials)

^b^Member of the National allergy society only

^c^Psychologist (n = 3, 0.3%), physician assistant allergy (n = 1, 0.1%), nurse practitioner in training (n = 2, 0.2%), resident doctor in training (n = 2, 0.2%), research associate (n = 3, 0.3%); health visitor (n = 2, 0.2%), medical student (n = 1, 0.1%)

^d^Paediatric respiratory doctor (n = 20; 1.7%); psychologist (n = 3;0.3%); tabacology (n = 1;0.1%); sports medicine (n = 2;0.2%); safeguarding (n = 1;0.1%); research associate (n = 2; 0.2%); public healthcare (n = 2;0.2%); pharmacology (n = 1; 0.1%); infectionist (n = 3; 0.3%)

^e^Participants were allowed to select more than 1 answer

### Resources

The majority (51%) of HCP’s consultations with AYA usually lasted about 20 min or less. Half of responders reported that patients had direct access to an allergy nurse and about 40% to either allergist, pulmonologist, dermatologist or gastroenterologist. Availability of social workers and psychologists was mostly lacking (18% and 24% respectively) (Table 2).

**Table 2:** Consultation

Practice parameters (n = 1179)	Number (%) respondents
HCPs category based on patient’s age^a^	
Paediatric	449 (38.1)
Adult	88 (7.5)
All ages groups	642 (54.5)
Time for follow-up consultation with AYA, minutes	
Up to 10	135 (11.5)
Up to 20	460 (39.0)
Up to 30	395 (33.5)
Up to 45	143 (12.1)
> 45	46 (3.9)
Direct access to healthcare professionals^b,c^	
Allergy/asthma nurse	597 (50.6)
Dietician	379 (32.1)
Paediatric allergist	537 (45.5)
Adult allergist	437 (37.1)
Psychologist	293 (24.9)
Respiratory physiotherapist	279 (23.7)
Social worker	209 (17.7)
Gastroenterologist	426 (36.1)
Pulmonologist	543 (46.1)
Dermatologists	502 (42.6)
Otolaryngologist	329 (27.9)
Referral only	42 (3.6)
Others^d^	4 (0.3)
Is care for AYA in your service organised differently than services to care for other age groups?	
No, specific resources	906 (76.8)
Yes, for all AYA	207 (17.6)
Yes, for selected patients only^e^	66 (5.6)
Percentage of AYA transferred to adult services rather than being discharged to GP or no care:	
1–10%	117 (9.9)
10–25%	123 (10.4)
25–50%	89 (7.5)
50–75%	108 (9.2)
75–100%	99 (8.4)
Don’t know	167 (14.2)
No transfer of AYA into adult services	198 (16.8)
We see all ages	278 (23.6)
Do you know how many of your transfer patients regularly attend the adult clinic after referral:	
No	361 (30.6)
Yes, please specify the percentage^f^	111 (9.4)
NA, no transfer of patients into adult services	405 (34.4)
NA, we see all ages	302 (25.6)
Evaluation tools on whether AYA is ready to be sent to adult service^b^	
No evaluation tool, AYA transferred at a specific age	489 (41.5)
Patient consent	171 (14.5)
Parental consent	122 (10.3)
Checklist of questions/knowledge	50 (4.2)
Completion of adolescent transition tool	48 (4.1)
We see all ages	364 (30.9)
My clinic does not transfer AYA to adult services	157 (13.3)
Feedback system between paediatric and local adult service^b^	
No system of feedback in place	569 (48.3)
The consultation letter from the first visit to adult clinic is sent back to referring paediatrician	150 (12.7)
Regular meetings to discuss patients	101 (8.6)
Not applicable, we see all ages	405 (34.4)

*AYA* adolescent and young adult, *GP* general practitioner, *HCP* healthcare professional, *NA* not applicable

^a^Paediatric HCP looking after 0–18 years old patients; adult HCP looking after ≥ 18 years old patients

^b^Participants were allowed to select more than 1 answer

^c^Direct access- without the referral from HCP

^d^Other: play therapist, family doctor trained in allergy, health visitor, immunologist

^e^Data is shown only for 31 (2.8%) responses: adherence problems (n = 1); educational sessions for asthma or peanut allergic patients (n = 1); AYA asthma clinic (n = 16); severe or multiple allergies (n = 10); referred to youth service (n = 1); need transition to adult allergy service and not to GP (n = 3); some have more time (n = 1); psychiatric problems (n = 2); school problems (n = 1); joint consultation with paediatric and adult allergist (n = 1); deprived backgrounds (n = 1)

^f^Data is only provided for 58 responses (%): median (LQ,UQ): 62.5 (37.5, 80); minimum 1; maximum 95

Notably, a total of 906 (77%) responders indicated that they had no specific resources to organize the care for AYA with allergy and asthma differently than services to care for other age groups. Specific resources such as e-learning materials (7.5%), workshops (7.1%), peer support (5.3%), phone hotline (4.7%) or webinars (2.8%) were rarely offered (Table 3). The availability of specific resources varied significantly between countries (p < 0.001 for no available resources, Additional file 1: Table S2). The lack of such resources was cited amongst the barriers to a satisfactory transition in comments from respondents (Box 1).

**Table 3:** Resources and other clinic elements to support adolescents and young adults with allergy and asthma in the medical services across Europe

Resources	N (%)
No specific resources	906 (76.8)
Consultation without parents present	300 (25.4)
Consultation letters are sent to paediatric or adult colleagues involved in individual patients’ care	289 (24.5)
Communication (emails, texts) addressed directly to the AYA (e.g. medical reports, appointments)	193 (16.4)
Transition report	165 (14.0)
Transition guideline for healthcare professionals	165 (14.0)
Joint transition clinics with the paediatric and adult services	104 (8.8)
Regular meetings involving paediatric and adult services in the field of allergy and pneumology	103 (8.7)
e-Learning materials	88 (7.5)
Workshops	84 (7.1)
Transition readiness assessment tool	64 (5.4)
Peer learning/peer support for patients	63 (5.3)
Phone hotline	56 (4.7)
Transition lead	48 (4.1)
Transition network	38 (3.2)
Webinars	33 (2.8)
Others^a^	12 (1.0)

*AYA* adolescent and young adult. Participants were allowed to select more than 1 answer

^a^Others: allergy nurse in the transition clinic; discussion about the transition process, adult clinic and self-management; disease- specific leaflets; referral to support groups/psychologist; email hotline; quality of life questionnaire; annual follow up. Results based on data from 1179 respondents

### Timing of transition

**Fig. 1:**
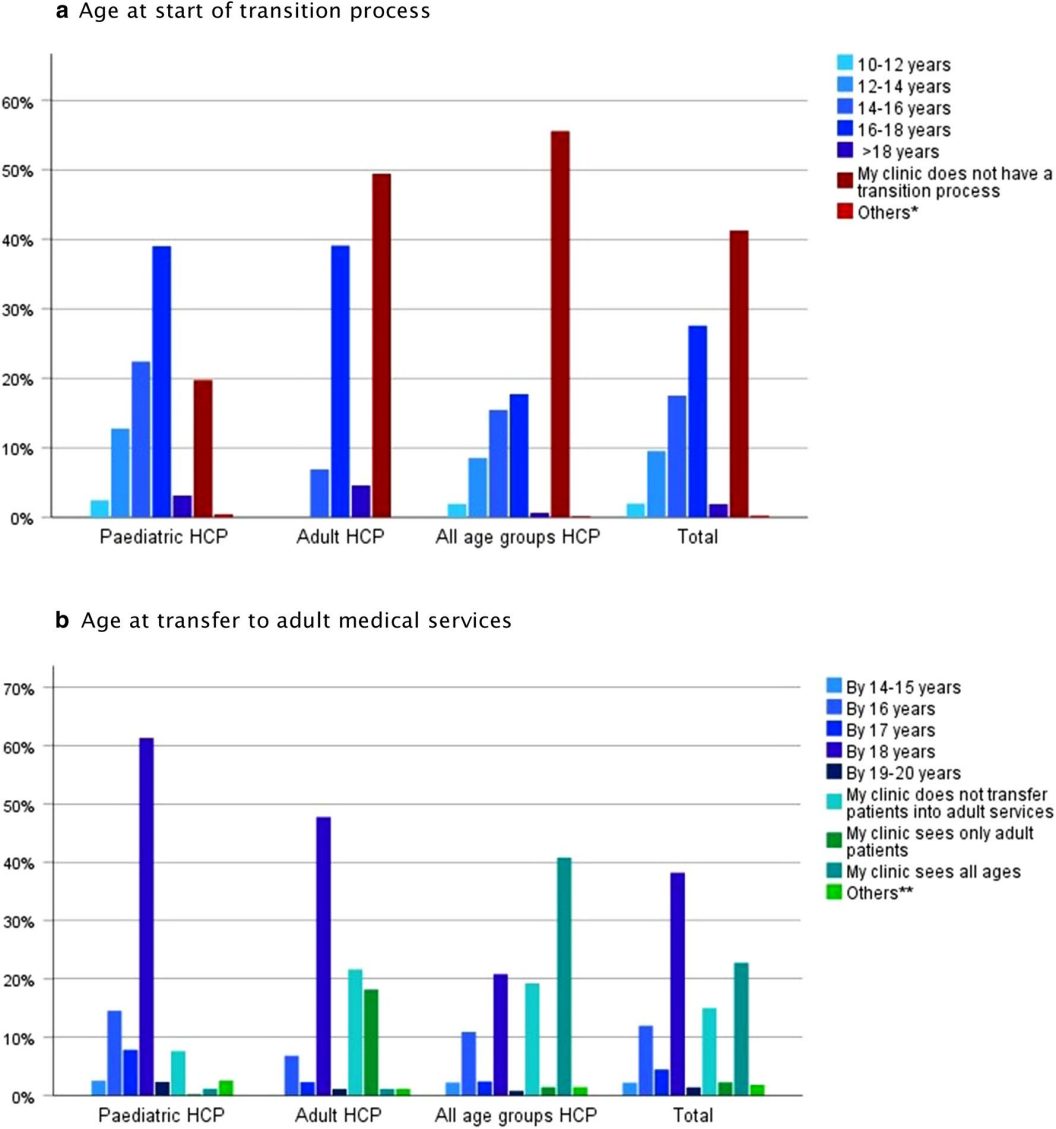
Age of adolescents and young adults with allergy and asthma when healthcare professionals start the transition process and transfer to adult medical services. HCP, healthcare professionals; Paediatric HCP (n = 449) looking after 0–18 years old patients; adult HCP (n = 88) looking after ≥ 18 years old patients; all ages groups HCP (n = 642). 1179 participants contributed to the statistical analysis. * Depending on the developmental stage and readiness. ** Depending on their secondary school graduation; after school or after university; based on the needs, readiness, developmental status of AYA, provider choice/availability

Overall, “My clinic does not have a transition process” was chosen by 20% paediatricians, 50% HCPs seeing only adults and 56% of those seeing all patients groups (Fig. 1). Twenty-eight percent of HCPs indicated that they started preparing AYA for transition at about 16–18 years. There were significant differences between countries (p < 0.001, Additional file 1: Table S2) in age of start of transition process, with significant interactions between clinic type and countries (Additional file 1: Figure S1). Almost 40% transferred AYA by the 18th year of age (Fig. 1).

### Approach to transition

The structure of the transition process varied across European countries (e.g. p < 0.001 for no specific resources, Additional file 1: Table S2). One-quarter of HCPs reported that they asked AYA whether they wanted to have a consultation without parents present, while only 16% of total sent medical-related correspondence directly to the AYA (Table 3). Less than 10% of HCPs had an established joint transition clinic with the paediatric and adult services or regular meetings to discuss individual cases. A mere 14% of respondents had a transition guideline for their service; 4% had a transition lead to oversee and coordinate the transition process and only 8.3% reported that they used a transition assessment tool or checklist of questions to determine transition readiness.

HCPs said that not all AYA were transfered to a specialist adult services. For example, only around half of those with poorly controlled asthma or on biological therapy were transfered (Additional file 1: Table S3). Among all responses, 30.6%) HCPs did not know whether their AYA patients attended the adult clinic after referral. (Table 2). Furthermore, nearly half of respondents (48%) reported a lack of an established feedback system between paediatric and local adult medical services after the AYA transferred care. Only thirteen percent identified that a medical report was sent from adult clinic to the referring paediatrician and only 9% discussed patients at a regular meeting between services (Table 2). There were substantial differences between countries in terms of feedback (p < 0.001 for all, Additional file 1: Table S2). Specific comments about approach to transition are summarised in Box 1.

### Training and challenges for healthcare professionals

**Fig. 2:**
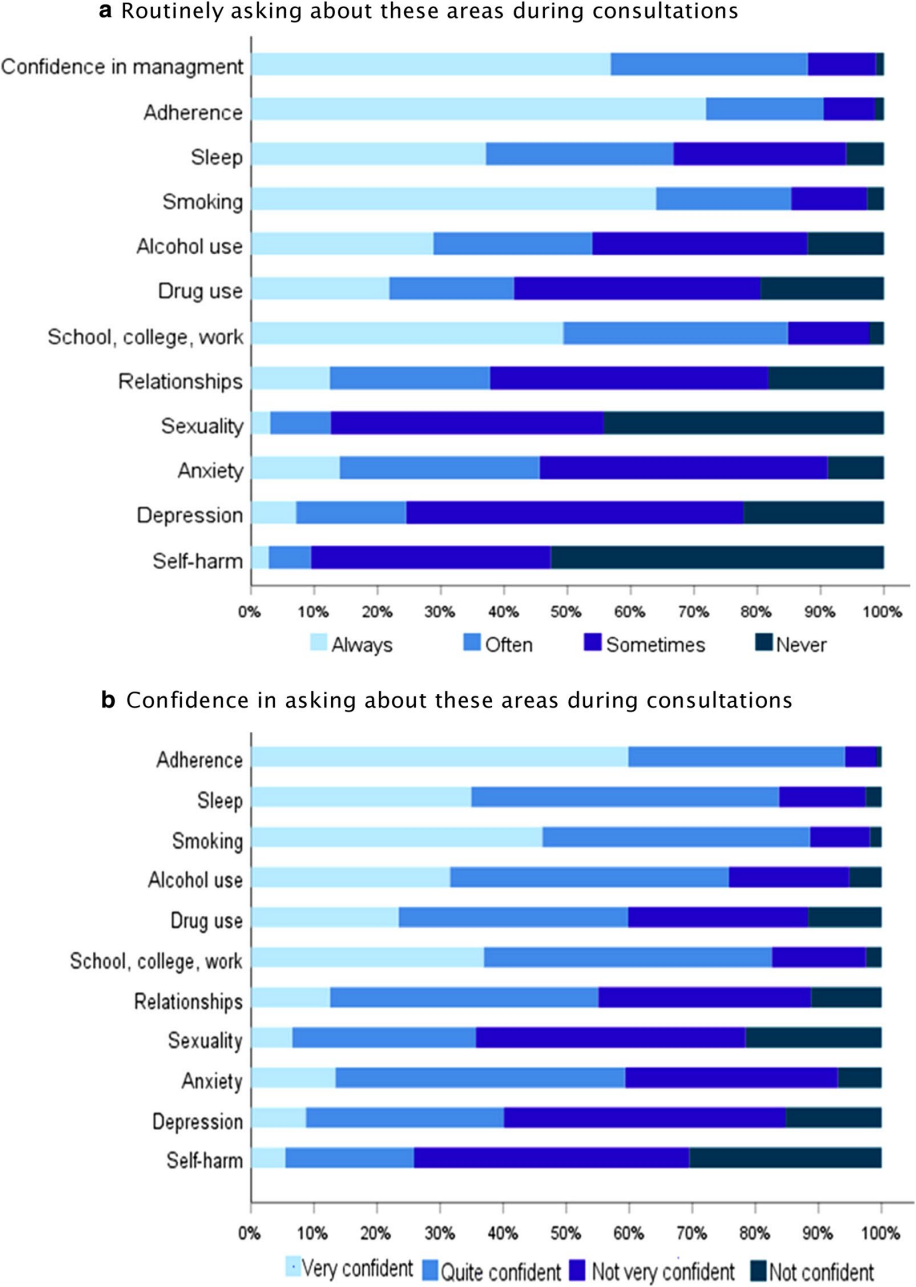

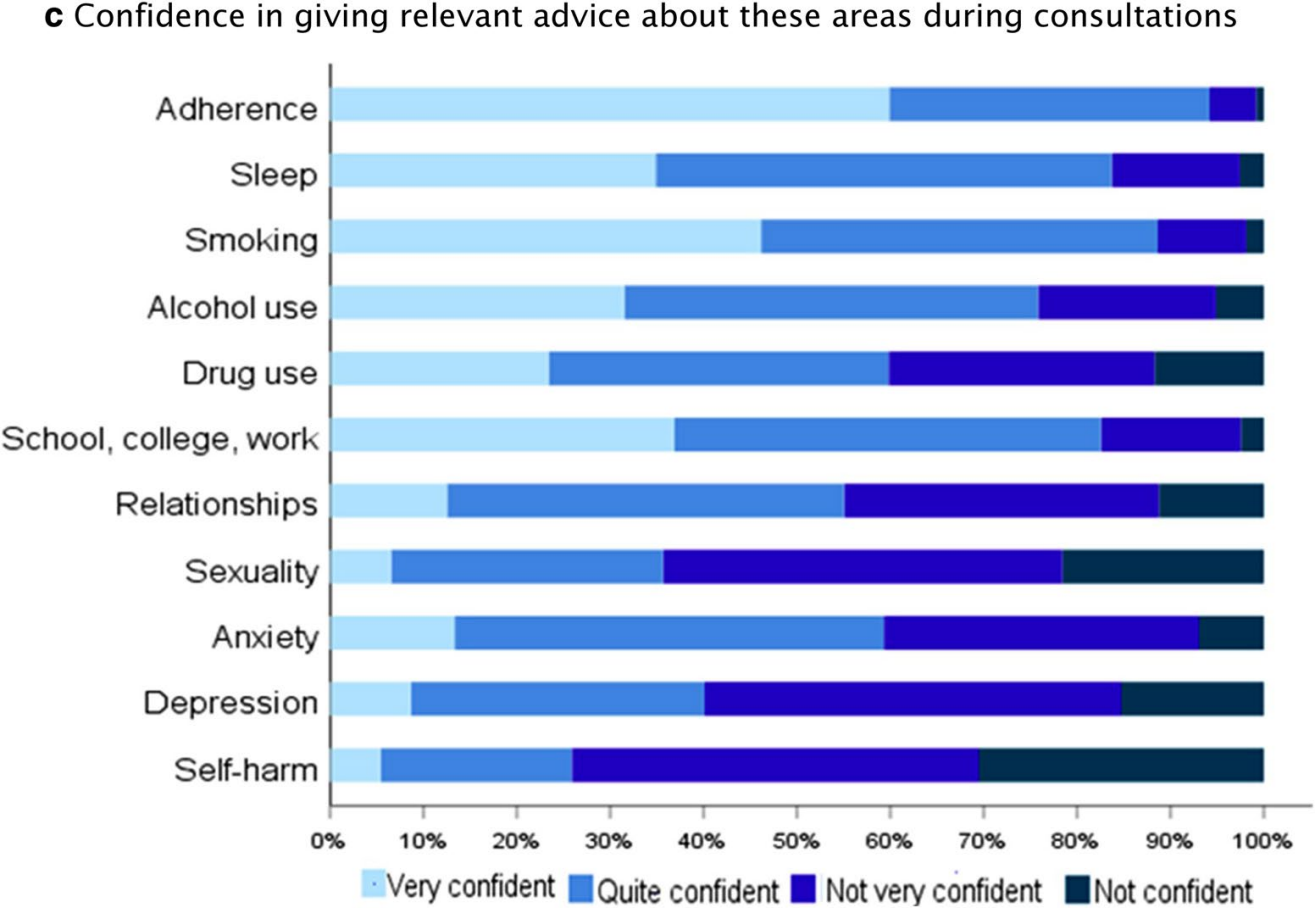
Challenges for healthcare professionals across Europe when managing adolescents and young adults with allergy and asthma. Results for each based on data from 1179 respondents. Additional file 1: Figure S6 demonstrates that respondents from different clinic types are similarly likely to ask about each area

A large proportion of HCPs never routinely asked about self-harm, sexuality, depression or drug use (Fig. 2, Additional file 1: Table S2). There was the same pattern of responses regarding confidence in asking and giving relevant advice about these areas. For example, HCPs were not very confident and not confident in asking about self-harm (66.6%), sexuality (64%), depression (43.6%) and drug use (41.5%). Some respondents commented specifically about importance of open dialogue with AYA (Box 1).

Seventy-six percent of all HCPs reported that they had not received specific training in the care of AYA (Additional file 1: Figure S3). Although respondents from clinics for all age groups, compared to paediatric ones, were more likely to have specific training (adjusted regression coefficient 0.033; 95% confidence interval 0.004, 0.062; p = 0.027), this also varied significantly by country (Additional file 1: Figure S3, Table S4). Box 1 summarises respondents’ comments concerning training.

### Importance of the transition care

Eighty-seven percent reported that they “strongly agree” or “agree” with the statement that transition is important for AYA with allergies and asthma (Additional file 1: Figure S5). Of the paediatric HCPs, 64% “strongly agree” with the statement while almost 50% of adult HCPs and HCPs looking after all ages groups chose this answer. The degree to which respondents from paediatric clinics (compared to clinics for all age groups) were more positive about the importance of transition varied by country (Additional file 1: Table S5, Figure S6). Notably, only 17% stated that transition is a priority in their country (Additional file 1: Figure S5). Specific comments from respondents about the importance of transition care are summarised in Box 1.

### Preconceptions and comments about transition process

Some HCPs thought that transition should happen even if AYA was not moving between medical services. However, others commented that they believed that there was no need for transition if HCPs cared for all ages of patients (Box 1).

Box 1. Example of comments from respondents **A. Adolescent and young adult-centred transition**

Communication

*‘An open dialog with the patient and his/her relatives is important as well as involving the patient in the treatment decision and plan.’*

*‘Engage them, tell them what is important, why it is important, how to recognise if things are not working. Give them control in the process. Understand their currencies (what is important to them). Let them be part of their roadmap.’*

*‘We should find appropriate communication methods for the Z generation.’*

*‘As patients grow up, we involve them more in their health issues and we try and find a time to speak to them without their parents present.’*

*‘It’s very important to take into account psychophysiological characteristics of AYA, their behavioural and social characteristics in order to make a personalised treatment plan.’*

*‘During this process, consulting a psychologist who is specialized in treating adolescents, should be proposed easily.’*

**B. Barriers for implementation of transition**

Lack of time

*‘Important but difficult to establish in busy work environment without more resources.’*

*‘I do not have time within my allergy clinic appointment to offer a full adolescent service.’*

Lack of resources

‘*Would dearly love to have a robust transition service* - *under*-*resourced and too many other competing priorities.’*

*‘Plenty of opportunity to improve, but requires resources.’*

*‘The importance of educating young adults about their conditions is underestimated however this is imperative to help them manage their condition, minimise risk and prevent attacks/anaphylaxis.’*

Not enough adult allergy specialists

*‘Adult services need funding, otherwise there is nowhere to transition the majority to. There is no dietitian in the adult services at my trust.’*

*‘Most paediatric secondary care allergy services don’t have a secondary care service to transition too. There is also nowhere to transition young people with multiple atopic comorbidities.’*

*‘We have no adult service for allergy within our hospital.’*

*‘Not enough adult allergists; not enough time spent for discussion with these patients.’*

Lack of training

‘*Never heard of it before.’*

*‘This survey has made me realize that I need to learn more about the transition process.’*

*‘Transition process should be known and educated.’*

*‘Very important if there are dedicated specialists. We have none in my country.’*

*‘Every doctor should have a training on this transition process.’*

*‘The problem is to think that allergic patients have to be seen by different specialists at different ages but perhaps we should have additional training to clinically evaluate patients of certain ages (children, teens) where some specialists may feel less comfortable.’*

*‘Doctors don’t have specific training in adolescence medicine.’*

**C. Importance of transition**

*‘Vitally important and should be addressed.’*

*‘Should be widely available.’*

*‘Should be implement in all clinic seeing allergy patients.’*

*‘The transition process is very important, we are currently working on a special transition program in our hospital.’*

*‘Disheartened we don’t have one.’*

*‘It is probably a luxury! There is so little basic allergy service for adults/children in our area that I feel they needs to be sorted first. Although I agree effective transition is very important abs would hugely help.’*

*‘We are in a process where we plan to do transition a high priority.’*

*‘It is critical that comprehensive and age appropriate services are developed for adolescence as this is the age group where they are most likely to be at risk, particularly if they have life threatening allergy. More needs to be done for this age group.’*

*‘It is very important transition because this category of patients is not very compliant…’*

*‘Transition process should be kept on high level of awareness.’*

*‘It is very important, we should pay higher attention.’*

**D. Transition protocol**

*‘There should be alignment across practices, health systems, countries.’*

*‘There is a lot of talk about addressing it, but very little opportunity for clinicians to get it right.’*

*‘I will be very happy if our colleges start to think about this process and if we change our practice in order to create and implement transition protocol.’*

*‘You highlighted a very important problem. I hope that these kind of questionnaires will made other doctors think more about this problem, made them do more for this particular group of patients so hopefully after the survey you will be able make a special guideline, algorithm to treat this group of patients better.’*

**E. Structure of transition**

Transition clinic

*‘A stand alone service for 16*–*25* *year olds would be very useful.’*

*‘Consider adolescence extending to age 25 with transitional clinic’.*

*‘It is a good thought in setting up special service for transition process.’*

Transition lead

*‘Needs dedicated lead and feedback process. Thus one does have the opportunity for meetings between team members on specific cases.’*

*‘All Trusts should employ a dedicated whole time specialist nurse to oversee and support the transition process and ensure that clinicians are supported during process too.’*

Multidisciplinary team, and joint clinics, feedback between paediatric and adult services

*‘I don’t transition patients to adults’ medical service but I think it’s very important to have continuity of the care and collaborations between medical professionals.’*

*‘Ideal for confidence: follow up by a mixed team child/adult.’*

*‘In many cases, a multidisciplinary psychological approach would be advisable.’*

*‘It’s very important to have system in place where pediatric and adult doctors collaborate with each other to maintain continuity of the medical care.’*

*‘The lack of collaboration between the specialties of pediatrics and immunoallergy interferes in a negative way in the follow*-*up and orientation of adolescents.’*

*‘It is important that the teen feels safe, confident and emphathizes with the doctor who has seen him and with the one who will see him going forward, so that adherence to treatment and other measures are appropriate. This is achieved with a good flow between paediatric pneumologist/allergists and adult allergists.’*

**F. Preconceptions about transition**

Transition should happen even if AYA is not moving between medical services

*‘The transition process is important for each patient. Even if they are not moving to adult care. Transition is a process of patient learning and self care.’*

*‘Sometimes clinicians think transition is the process of moving between paediatric and adult services rather than discharging back to primary care. The importance of educating young adults about their conditions is underestimated however this is imperative to help them manage their condition, minimise risk and prevent attacks/anaphylaxis.’*

*‘I think transition to adult services is less important than a transition to adult management of their allergies. I.e. the transition is about reinforcing their independent management rather than about moving them to adult clinics.’*

There was no need for transition if a HCP sees all ages of patient

*‘If the same physician takes care of allergic patients from 0 to 100* *years, there is no need for transition and the physician who knows better the disease state of the patient can decide whether it is recommendable to discharge or to continue the follow*-*up.’*

*‘In an allergy service where patients are seen throughout their whole life this problem is sorted.’*

*‘There is no transition. We see how the patient progresses as a whole from infancy.’*

*‘Allergists should be the specialists who see allergic patients regardless of their age and then there would be no problem with transition.’*

*‘The transition issue does not apply if patients of all ages are seen in a department in a suitable setting.’*

*‘Not applicable for my praxis, we treat and deal our paediatric patients continuously till adulthood.’*

Seeing the same doctor is important

*‘Is important to have same doctor because he knows more well your history.’*

*‘I believe that the best allergy care system is one where an allergist will patient all the time!’*

AYA, adolescent and young adult; HCP, healthcare professionals. Healthcare professionals’ comments were summarised using a qualitative data analysis approach. Text was divided into separate units, coded and summarized as themes in duplicate.

## Discussion

This is the first survey on AYA with allergies and asthma aiming to provide an insight into the current reality of transition practices of frontline HCPs across Europe. Although most respondents felt that transition care was important, only a minority had a transition process or policy in place, as per the National Institute for Health and Care Excellence guideline (NICE) in the UK [20] or the Children and Young’s People Allergy Network Scotland (CYANS) Transition Pathway in Scotland [21]. Moreover, transition care varies significantly by country and clinic type but it usually started late in adolescence. A small proportion of respondents had dedicated or specific resources for delivering transition care. Most did not see AYA alone for part of the consultation. There was often minimal liaison between paediatric and adult primary care and/or specialist services. The lack of specific training around AYA and the lack of transition guidelines for this group may be driving these significant limitations and variations in care.

According to the results of the survey, many allergy services do not have a transition process to support adolescents to become independent patients. Comments from respondents suggest that many HCPs do not believe that transition is required when a clinic or service sees all age groups. However, all patients go through the similar developmental stages and require support and education in self-management of the disease despite staying within the same department. Where transition processes exist, they mostly start at 16–18 years and patients are transferred by age 18. It has been argued that preparations for transition should be initiated early, [8, 18, 19] around 11–13 years, to allow the development of self-management skills and optimise other health and well-being outcomes. Facilitation of independence in the children’s department is a vital step that prepares AYA to take responsibility for their lives and health prior to transition to adult services. Successful transition practices depend on the AYA’s developmental stage; thus HCPs should enable AYA to gradually take a leading role [8, 18]. Unfortunately, this survey shows that only 25% of HCPs have any consultation with the AYA without parents and only some addressed medical communication directly to AYA.

In many European countries the timing of transition of AYA from paediatric to adult care is determined by the patient’s chronological age (usually 18 years) rather than based on individual and patient-centred AYA readiness. Only 4.2% of HCPs reported that they use a questionnaire assessment to determine readiness for transition. Therefore, there is a need to help guide HCPs to initiate transition when AYA are developmentally ready and nurture self-management skills. There are several generic instruments such as the ‘Transition Readiness Assessment Questionnaire’ [22] (TRAQ), or ‘Ready Steady Go’ [23] that could be used to regularly access transition readiness. For instance, TRAQ has been shown to be a useful tool in measuring skills needed for successful transition in AYA with special health care needs and guiding educational interventions to support transition in different areas of life such as education, work and daily life [22].

Communication with AYA is key for smooth and successful transition [15]. This should include holistic discussions about the disease, promotion of independence and self-management skills as well as other important areas of AYAs’ health and well-being. The HEADSS (Home, Education/Employment, peer group, Activities, Drugs, Sexuality, Suicide/depression) assessment has been successfully implemented in clinical practise to facilitate effective communication with AYA [24]. The results of this survey revealed that discussions about self-harm, sexuality, depression and drug use are mostly lacking in the majority of consultations with AYA with allergic diseases. HCPs have little confidence in asking and giving relevant advice about these areas despite self-harm and depression being important co-morbidities in allergies and asthma [11].

To deliver a successful transition process, a multidisciplinary approach and feedback between paediatric and adult medical services are required [15]. This survey revealed that only a few clinics have social workers or psychologists available to help address transition-relevant issues. Given that allergic diseases interact with psychological factors and are associated with increased anxiety, depression and suicidal thoughts [25–27] there is a need for an investment in training for HCPs in recognising mental health problems and direct access to specialists to address these needs. Poor communication between paediatric and adult clinicians was also identified by this survey. For instance, only 13% stated that they routinely sent a transition report, similar to those reported by adult endocrinologists, who identified it as a key barrier for successful transition. [28] In this survey even fewer (8.8% HCPs) reported they had a joint transition clinic with the AYA, his or her family, paediatric and adult HCPs; although a joint clinic is recommended in many disease-specific transition guidelines [29]. Preventing patients becoming lost between paediatric and adult services has been identified as a major challenge for HCPs [30]. A transition lead who can coordinate and facilitate communication could be helpful [8, 18, 20] but is currently lacking (95.9% stated they did not have one).

Overall, differences in transition practices could be explained by the lack of training, dedicated resources and a guideline in the care of AYA with allergies and asthma. A study of AYA with diabetes showed that a transition programme that consisted of disease education, case management, transition clinic, transition website and group classes improved adherence to follow up and health outcomes in comparison with usual care [31]. Several key components of the training in generic components of transition have already been proposed in rheumatic diseases transition guidelines [18] and could be adjusted for HCPs working in the allergy field. There was a strongly positive reply from the survey respondents on the importance of transition for AYA with allergies and asthma which highlights the need to develop transition programmes for these patients.

### Strengths and limitations of the survey

The survey was developed to be European representative; although there were several limitations. Firstly, it was limited to HCPs with membership of either EAACI or NAS, which could have caused selection bias. Secondly, it was not possible to obtain the number of members from each NAS to calculate the overlap with EAACI membership and therefore the precise response rate. Thirdly, those who did not participate might have different transition practices than the respondents of the survey. However, the results highlight the discrepancy and unmet need in transition care for AYA with allergies and asthma across Europe. The representativeness of the survey is likely to be high given the large number of HCPs who responded across Europe with good representation across countries, specialities, work settings and levels of experience. However, some countries were overrepresented which could potentially shift the overall results toward current practices in Italy, Russia, Spain and the United Kingdom. In addition, the response from some countries was too low to draw any national conclusion.

### Implications

These survey results have important implications. The survey highlights deficits in current transition practice for AYA with allergy and asthma and the lack of specific training for HCPs in the care of this age group. Specific asthma and allergy ‘readiness to transition’ tools are not being used despite being able to ensure transition support is available at the developmentally appropriate time for individual patients. Further steps must focus on the development of evidence-based recommendations and standardization of the transition of care relevant to the needs of these patients. This should be agreed on a European level, acknowledging possible national differences in health care systems. It is hoped that a structured transition program will lead to improvements in patient knowledge, compliance, self-efficacy and self-management. Furthermore, training in the generic transition process should be implemented in undergraduate and postgraduate training programmes. Lastly, these findings should focus policy makers on the need to invest in planning these transition services and appropriately resourcing them.

## Conclusions

This survey demonstrates significant limitations and variations in the delivery of transition practices for AYA with allergies and asthma in Europe. These findings should be used as a catalyst for standardisation and harmonisation of the delivery of transitional care across European countries to facilitate successful transition, improve well-being and healthcare outcomes of these patients.

## Supplementary information

**Additional file 1.** Additional tables and figures.

## Data Availability

The datasets used and/or analysed during the current study are available from the corresponding author on reasonable request.

## Abbreviations

AYA: Adolescents and young adults
HCP: Healthcare professionals
EAACI: European Academy of Allergy and Clinical Immunology
NAS: National Allergy Societies
NICE: National Institute for Health and Care Excellence guideline
CYANS: Children and Young’s People Allergy Network Scotland
TRAQ: Transition Readiness Assessment Questionnaire
HEADSS: Home, Education/Employment, Activities, Drugs, Sexuality, Suicide/depression
