# Supplementary material for: Current transition management of adolescents and young adults with allergy and asthma: a European survey
Source: Clin Transl Allergy. 2020 Oct 7;10:40. doi: 10.1186/s13601-020-00340-z (PMC7542112; doi:10.1186/s13601-020-00340-z)
Supplement: Supplementary file 1 — Additional file 1. Additional tables and figures. [file 13601_2020_340_MOESM1_ESM.pdf]

Additional file

Title: Current transition management of adolescents and young adults with allergy and asthma: A European survey.

Authors: Ekaterina Khaleva[1], Marta Vazquez-Ortiz[2], Pasquale Comberiati[3,4], Audrey DunnGalvin[5,6], Helena Pite[7,8], Katharina Blumchen[9], Teresa Garriga-Baraut[10,11], Valerie Hox[12], Alexandra F. Santos[13,14,15], Claudia Gore[2,16], Rebecca C Knibb[17], Cherry Alviani[1,18], Charlotte G. Mortz[19], Elizabeth Angier[20], Bettina Duca[16], Britt Jensen[19], Silvia Sanchez-Garcia[21], M. Hazel Gowland[22], Frans Timmermans[23], Oliver Pfaar[24], Graham Roberts[1,18,25].

Affiliations:

1. Faculty of Medicine, University of Southampton, Southampton, UK.

2. Section of Inflammation, Repair and Development, National Heart and Lung Institute, Imperial College London, UK

3. Department of Clinical and Experimental Medicine, Section of Paediatrics, University of Pisa, Pisa, Italy

4.Department of Clinical Immunology and Allergology, I.M. Sechenov First Moscow State Medical University, Moscow, Russia.

5. Applied Psychology and Paediatrics and Child Health, University College Cork, Cork, Ireland

6. Paediatrics and Child Infectious Diseases, First Moscow State Medical University, Moscow, Russia.

7. Allergy Center, CUF Descobertas Hospital and CUF Infante Santo Hospital, Lisbon, Portugal

8.CEDOC, Chronic Diseases Research Center, NOVA Medical School/Faculdade de Ciências Médicas, Universidade Nova de Lisboa, Lisbon, Portugal

9. Department of Paediatric and Adolescent Medicine, Paediatric Pneumology, Allergology and Cystic Fibrosis, University Hospital Frankfurt, Frankfurt am Main, Germany

10. Unitat d'Allergologia Pediàtrica, Hospital Universitari Vall d'Hebron, Barcelona, Spain

11. Grup d’Investigació “Creixement i Desenvolupament”, Institut de Recerca de l’Hospital Universitari Vall d’Hebron (VHIR), Barcelona, Spain.

12. Department of Otorhinolaryngology, Head and Neck Surgery, University Hospitals Saint-Luc, Brussels, Belgium.

13. Department of Women and Children’s Health (Paediatric Allergy), School of Life Course Sciences, Faculty of Life Sciences and Medicine, King’s College London, London, UK

14. Peter Gorer Department of Immunobiology, School of Immunology and Microbial Sciences, King’s College London, London, UK

15. Children’s Allergy Service, Guy’s and St Thomas’ Hospital, London, UK; Asthma UK Centre in Allergic Mechanisms of Asthma, London, UK

16. Department of Paediatrics, Imperial College Healthcare NHS Trust, London, UK

17. Department of Psychology, School of Life and Health Sciences, Aston University, Birmingham, UK

18. The David Hide Asthma and Allergy Research Centre, St Mary's Hospital, Isle of Wight, UK

19. Department of Dermatology and Allergy Centre, Odense Research Centre for Anaphylaxis (ORCA), Odense University Hospital, University of Southern Denmark, Kloevervaenget 15, DK-5000 Odense C, Denmark

20. Primary Care and Population Sciences, University of Southampton, Southampton, UK

21. Allergy Department, Hospital Infantil Universitario del Niño Jesús, Madrid, Spain

22. Allergy Action, St Albans, UK

23. Nederlands Anafylaxis Netwerk – European Anaphylaxis Taskforce, Dordrecht, The Netherlands

24. Department of Otorhinolaryngology, Head and Neck Surgery, Section of Rhinology and Allergy, University Hospital Marburg, Philipps-Universität Marburg, Marburg, Germany

25. NIHR Southampton Biomedical Research Centre, University Hospital Southampton NHS Foundation Trust, Southampton, UK.

Address for correspondence:

Professor Graham Roberts, Paediatric Allergy and Respiratory Medicine, University Child Health (MP803), University Hospital, Southampton NHS Foundation Trust, Tremona Road, Southampton SO16 6YD, UK.

Tel.: +44 (0) 2380796160 E-mail: g.c.roberts@soton.ac.uk

Contents

Title page	1
Contents	3
Questionnaire	4
Number of participants from non-European countries	14
Table S1. Sensitivity analysis of the key questions per language	15
Table S2. Comparison of transition practices in seven European countries with more than 50 responses	16
Figure S1. Age at a start of transition process based on clinic type in seven countries with more than 50 responses	18
Table S3. Criteria for transition of adolescents and young adults with allergy and asthma to adult medical services in Europe	19
Figure S2. Challenges for HCPs based on clinic type in Europe. Do you routinely ask about the following areas?	20
Figure S3. Specific training in adolescents and young adults transition process	21
Figure S4. Have you had any specific training in the care of adolescents and young adults such as dedicated training course, online training course or supervision within clinic?	22
Table S4. Specific training in management of adolescent and young adults with allergy and asthma based on type of clinic in 7 countries	23
Figure S5. Agreement of healthcare professionals with the statement: “transition is important for adolescents and young adults with allergies or asthma’’	24
Figure S6. Agreement of healthcare professionals with the statement: “transition is important for adolescents and young adults with allergies or asthma’’ based on clinic type in countries with more than 50 responses	25
Table S5. Agreement of healthcare professionals with the statement: “transition is important for adolescents and young adults with allergies or asthma’’ based on clinic type in countries with more than 50 responses	26
Figure S7. Does the department of health in your country make transition process a high priority?	27

Survey title: How do you manage “terrific” or “terrifying” teens (11-25 years)? Win an Amazon Voucher!

Adolescence is an important phase of life as children develop into adults. Paediatric patients need to learn about their allergies and asthma and how to successfully self-manage them. Adolescents and young adults (i.e. patients aged 11-25 years old) may have challenges in self-management that are different to other age groups.

The EAACI Task Force on Adolescents and Young Adults is aiming to develop advice for healthcare professionals to help them provide support in self-management for adolescents and young adults. As a first part of this activity, we would like to invite you to complete a short survey (10 minutes) to allow us to understand the current state of adolescent practice.

Glossary:

Transition is a process of empowering adolescent patients into becoming competent adult patients.

Transition readiness assessment tool is a questionnaire completed by the patient or healthcare professional (e.g. ‘ready, steady, go’ or hospital’s own document)

Transition network means named paediatric and/or adult colleague(s) in an area with an interest in adolescent and young adults transition process

Transition report is a detailed written letter to adult colleague summarising the paediatric care

Adolescents and young adults are defined as patients with allergies or asthma aged 11-25 years

1. Which EAACI Section/Interest Group are you affiliated with? (tick just one)
    - Asthma
    - Dermatology
    - ENT
    - Immunology
    - Pediatrics
    - Primary Care and Allied Health
    - None

2. In which country do you work?
    - Austria
    - Belarus
    - Belgium

- Bulgaria
- Croatia
- Cyprus
- Czech Republic
- Denmark
- Estonia
- Finland
- France
- Germany
- Greece
- Ireland
- Italy
- Latvia
- Lithuania
- Luxembourg
- Malta
- Netherlands
- Norway
- Poland
- Portugal
- Russia
- Romania
- Slovakia
- Slovenia
- Spain
- Sweden
- Switzerland
- United Kingdom
- Ukraine
- Other [please name]

3. What is your profession?

- Doctor
- Specialist allergy nurse
- Dietitian
- Psychologist
- Other (please state)

4. Please select your specialty (tick all that apply):
    - Paediatric allergy
    - Paediatrics
    - Allergy (adults only)
    - Allergy (children and adults)
    - Dermatology
    - Respiratory Medicine
    - ENT
    - General Practitioner
    - Other (please specify)

5. In which setting do you work? (tick all that apply):
    - Tertiary care (e.g. highly specialised teaching hospital)
    - Secondary care (e.g. hospital setting with some degree of specialisation)
    - Primary care (e.g. outpatient clinic, walk-in clinic)
    - Private practice
    - Other (please describe)

6. How long have you been working with patients with allergies and asthma?
    - Please state number of years

7. What age range does your service cover?
    - All ages
    - 0-14
    - 0-16
    - 0-18

- ≥ 12
- ≥ 14
- ≥ 16
- ≥ 18
- Other (please specify)

8. How much time do you have for your usual follow-up consultation with adolescents and young adults?
    - Up to 10 min
    - Up to 20min
    - Up to 30min
    - Up to 45min
    - >45min

9. Does your service have direct access (without being referred by a doctor or other healthcare practitioner) to the following healthcare professionals? Tick all that apply
    - Allergy / asthma nurse
    - Dietician
    - Paediatric allergist
    - Adult allergist
    - Psychologist
    - Respiratory physiotherapist
    - Social worker
    - Gastroenterologist
    - Pulmonologist
    - Dermatologists
    - Audiologists
    - Others (please specify)

10. When you see adolescents and young adults in your practice, **do you routinely ask** about the following areas (tick as appropriate):

	Never	Sometimes	Often	Always
Sleep				
Anxiety				
Depression				
Self-harm				
Smoking				
Alcohol use				
Drug use				
Relationships				
School/college/work				
Sexuality				
Adherence				
Confidence in managing their own allergies and/or asthma				

11. When you see adolescents and young adults in your practice, **how confident do you feel** to ask about the following areas (tick as appropriate):

	Not confident	Not very confident	Quite confident	Very confident
Adherence				
Sleep				
Anxiety				
Depression				
Self-harm				
Smoking				
Alcohol use				
Drug use				
Relationships				
School/college/work				
Sexuality				

12. When you see adolescents and young adults in your practice, **how confident do you feel that you can provide good/relevant advice** for the following areas (tick as appropriate):

	Not confident	Not very confident	Quite confident	Very confident
Adherence				
Sleep				
Anxiety				
Depression				
Self-harm				
Smoking				
Alcohol use				
Drug use				
Relationships				
School/college/work				
Sexuality				

13. Is the care for adolescents and young adults in your service organised differently than services to care for other age groups?

- No, we have no specific resources for this age group
- Yes, for all adolescents and young adults
- Yes, for selected patients only (please say which patients)

14. What resources do you have that are specifically for adolescents and young adults with allergies and asthma in your service? Tick all that apply

- Not applicable, we have no specific resources for adolescents and young adults
- Transition guideline for healthcare professionals
- Transition readiness assessment tool
- Workshops
- Peer learning/peer support for patients
- Webinars
- e-Learning materials
- Phone hotline
- Consultation without parents present (e.g. at certain age parents are asked to leave the consultation room and the patient seen independently)
- Joint transition clinics with the paediatric and adult services
- Transition lead

- Transition network
- Regular meetings involving paediatric and adult services in the field of allergy and pneumology to discuss adolescent and young adult patients
- Transition report
- Consultation letters or reports are sent to paediatric or adult colleagues involved in individual patients' care
- Communication (post, emails, texts) addressed directly to the adolescent or young adult (e.g. medical reports, letters, appointments)
- Other (please specify)

15. At what age do you usually **<u>start</u>** the transition process (i.e. adolescents' preparation to be followed up in an adult healthcare services)?
    - Not applicable, my clinic doesn't have a transition process
    - 10-12 years
    - 12-14 years
    - 14-16 years
    - 16-18 years
    - Other (please specify)

16. At what age are adolescents transferred to adult services in your service?
    - Not applicable, my clinic does not transfer patients into adult services
    - Not applicable, my clinic sees all ages
    - Not applicable, my clinic sees only adult patients
    - By their 16th birthday
    - By their 17th birthday
    - By their 18th birthday
    - Other (specify)

17. Please estimate what percentage of your patients are transferred to adult services rather than being discharged to either family doctor care or no care:
    - 1-10%
    - 10-25%
    - 25-50%

- 50-75%
- 75-100%
- Don’t know
- Not applicable, my clinic does not transfer patients to adult services
- Not applicable, we see all ages

18. Do you know how many of your transition patients regularly attend the adult clinic after referral:

    - Yes, please specify the percentage
    - No
    - Not applicable, my clinic does not transfer patients to adult services
    - Not applicable, we see all ages

19. Which criteria should an adolescent meet in your department to be transferred to the adult services rather back to primary care? Tick all that apply:

    - Not applicable, my clinic refers all patients to adult services
    - Not applicable, my clinic refers no patients to adult services
    - Not applicable, we see all ages
    - All paediatric patients with any food allergy
    - All paediatric patients with multiple food allergies
    - All paediatric patients with multiple food allergies and asthma
    - All paediatric patients with multiple food allergies, asthma and adrenaline autoinjector
    - All paediatric patients who have ever experienced anaphylaxis
    - All paediatric patients with food allergy who also have an adrenaline auto-injector
    - All paediatric patients on immunotherapy for respiratory allergy
    - All paediatric patients who have gone through immunotherapy for food allergy
    - All paediatric patients with asthma
    - All paediatric patients with difficult, severe or poorly controlled asthma
    - All paediatric patients on biologics for asthma or chronic spontaneous urticaria/angioedema
    - All paediatric patients with severe or uncontrolled atopic dermatitis
    - All paediatric patients with allergic rhinoconjunctivitis

- All paediatric patients with severe allergic rhinoconjunctivitis
- All paediatric patients with hymenoptera venom allergy
- All paediatric patients on immunotherapy for hymenoptera venom allergy
- Other (please specify)

20. How do you evaluate whether the patient is ready to be sent to adult services? Tick all that apply
    - We have no evaluation tool, patients transferred at a specific age
    - Parental consent
    - Patient consent
    - Checklist of questions/knowledge that the patient completes
    - Completion of 'ready, steady, go' or similar adolescent transition tool
    - Other (please specify)
    - Not applicable, we see all ages
    - Not applicable, my clinic does not transfer patients to adult services

21. Is there a feedback system between your paediatric service and your local adult service? (tick all that apply)
    - Regular meetings to discuss patients
    - The consultation letter from the first visit to the adult clinic is sent back to the referring paediatrician
    - No system of feedback in place
    - Not applicable, we see all ages

22. Have you had any specific training in the care of adolescents and young adults? (tick all that apply)
    - Dedicated training course
    - Online training course
    - Supervision within clinic
    - No specific training

23. Does the department of health in your country make transition a high priority:

- Yes
- No
- Don’t know

24. To what extent do you agree with the statement, transition is important for adolescent and young adult patients with allergies or asthma. (tick as appropriate):

Strongly agree	Agree	Neither agree nor disagree	Disagree	Strongly disagree	Not applicable

25. Please let us know about any other thoughts you have about the transition process.

Thank you for taking the time to complete this survey. The results will inform our understanding of current practice and ensure that the EAACI Taskforce’s advice covers the right areas to help your practice in the future.

To be entered into Amazon voucher draw, please leave your email in the box below which will be removed prior to analysis in order to maintain anonymity.

Ekaterina Khaleva, Marta Vazquez Ortiz and Graham Roberts on behalf of the EAACI Adolescent and Young Adult taskforce

Number of participants from non-European countries: Egypt (n=1; 0.1%), New Zealand (n=2; 0.2%), Philippines (n=2; 0.2%), Saudi Arabia (n=1; 0.1%), Brazil (n=11; 0.9%), Zimbabwe (n=1; 0.1%), India (n=5; 0.4%), Malaysia (n=1; 0.1%), Canada (n=6; 0.5%), USA (n=8; 0.6 %), Curacao (n=1; 0.1%), Qatar (n=1; 0.1%), Japan (n=2; 0.2%), South Africa (n=1; 0.1%), Australia (n=6; 0.5%), Nigeria (n=1; 0.1%), Thailand (n=2; 0.2%), Sri Lanka (n=2; 0.2%), Iran (n=1; 0.1%), Brunei (n=1; 0.1%), Chile (n=1; 0.1%), Paraguay (n=1; 0.1%), Indonesia (n=1; 0.1%), Mexico (n=10; 0.8%), Dominican Republic (n=1; 0.1%), Argentina (n=4; 0.1%), Honduras (n=1; 0.1%), Nicaragua (n=1; 0.1%).

Table S1. Sensitivity analysis of the key questions per language

	English (n=537)	Italian (n=105)	Portuguese (n=53)	Russian (n=204)	German (n=74)	Spanish (n=146)	Total (n=1119)	P value†	χ2 value	DF
**Transition is important for AYA with allergies/asthma, n (%)**								**<0.001**	63.1	
Strongly agree	266 (50.5)	63 (60.6)	16 (39.0)	135 (66.8)	33 (44.6)	71 (53.8)	584 (54.1)			
Agree	201 (38.1)	36 (34.6)	15 (36.6)	45 (22.3)	20 (27.0)	35 (26.5)	352 (32.6)			
Neither agree nor disagree	50 (9.5)	4 (3.8)	6 (14.6)	20 (9.9)	17 (23.0)	20 (15.2)	117 (10.8)			
Disagree	8 (1.5)	0 (0.0)	2 (4.9)	2 (1.0)	4 (5.4)	5 (3.8)	21 (1.9)			
Strongly disagree	2 (0.4)	1 (1.0)	2 (4.9)	0(0.0)	0(0.0)	1 (0.8)	6 (0.6)			
**Specific training in the care of AYA with allergies/ asthma, n (%)** [a]										
Dedicated training course	61 (11.4)	11 (10.5)	15 (28.3)	26 (12.7)	3 (4.1)	13 (8.9)	129 (11.5)	**0.001**‡	20.1	5
Online training course	24 (4.5)	2 (1.9)	0 (0.0)	11 (5.4)	0 (0.0)	7 (4.8)	44 (3.9)	0.133	7.9	
Supervision within clinic	80 (14.9)	7 (6.7)	13 (24.5)	31 (15.2)	7 (9.5)	19 (13.0)	157 (14.0)	**0.042**‡	11.5	5
No specific training	395 (73.6)	88 (83.8)	28 (52.8)	155 (76.0)	66 (89.2)	112 (76.7)	844 (75.4)	**<0.001**‡	27.3	5
**Feedback system between paediatric and adult service, n (%)** [a]										
No system of feedback in place	281 (52.3)	75 (71.4)	11 (20.8)	108 (52.9)	30 (40.5)	42 (28.8)	547 (48.9)	**<0.001**‡	67.7	5
Regular meetings to discuss patients	42 (7.8)	2 (1.9)	2 (3.8)	30 (14.7)	7 (9.5)	17 (11.6)	100 (8.9)	**0.001**	19.0	
The consultation letter from the first visit to the adult clinic is sent back to the referring paediatrician	111 (20.7)	4 (3.8)	1 (1.9)	13 (6.4)	12 (16.2)	4 (2.7)	145 (13.0)	**<0.001**‡	63.9	5
NA, we see all ages	127 (23.6)	24 (22.9)	39 (73.6)	66 (32.4)	32 (43.2)	86 (58.9)	373 (33.3)	**<0.001**	113.7	
**Evaluation of AYA in order to be sent to adult services, n (%)** [a]										
Patients transferred at a specific age	226 (42.1)	52 (49.5)	8 (15.1)	113 (55.4)	23 (31.1)	52 (35.6)	474 (42.4)	**<0.001**‡	39.1	5
Parental consent	75 (14.0)	14 (13.3)	2 (3.8)	6 (2.9)	9 (12.2)	10 (6.8)	116 (10.4)	**<0.001**‡	25.3	5
Patient consent	115 (21.4)	13 (12.4)	4 (7.5)	9 (4.4)	15 (20.3)	8 (5.5)	164 (14.7)	**<0.001**‡	51.0	5
Checklist of questions/knowledge that AYA completes	33 (6.1)	5 (4.8)	1 (1.9)	6 (2.9)	2 (2.7)	3 (2.1)	50 (4.5)	0.212	6.8	
Completion of adolescent transition tool	39 (7.3%)	2 (1.9)	1 (1.9)	1 (0.5)	2 (2.7)	2 (1.4)	47 (4.2)	**<0.001**	24.4	
NA, we see all ages	124 (23.1)	19 (18.1)	35 (66.0)	55 (27.0)	31 (41.9)	75 (51.4)	339 (30.3)	**<0.001**‡	89.2	5
NA, my clinic does not transfer patients to adult services	77 (14.3)	15 (14.3)	6 (11.3)	33 (16.2)	5 (6.8)	12 (8.2)	148 (13.2)	0.141‡	8.3	5
**Resources and other clinic elements to support AYA with allergies/asthma in your service, n (%)** [a]										
We have no specific resources for AYA	298 (55.5)	75 (71.4)	26 (49.1)	115 (56.4)	34 (45.9)	121 (82.9)	669 (59.8)	**<0.001**‡	51.8	5
Transition guideline for healthcare professionals	93 (17.3)	11 (10.5)	10 (18.9)	35 (17.2)	4 (5.4)	4 (2.7)	157 (14.0)	**<0.001**‡	28.6	5
Transition readiness assessment tool	54 (10.1)	2 (1.9)	0 (0.0)	4 (2.0)	2 (2.7)	1 (0.7)	63 (5.6)	**<0.001**	38.1	
Workshops	37 (6.9)	7 (6.7)	2 (3.8)	27 (13.2)	4 (5.4)	4 (2.7)	81 (7.2)	**0.009**	15.1	
Peer learning/peer support for AYA	30 (5.6)	7 (6.7)	3 (5.7)	11 (5.4)	6 (8.1)	5 (3.4)	62 (5.5)	0.737	2.7	
Webinars	16 (3.0)	4 (3.8)	1 (1.9)	5 (2.5)	5 (6.8)	0 (0.0)	31 (2.8)	**0.054**	9.9	
e-Learning materials	52 (9.7)	3 (2.9)	7 (13.2)	20 (9.8)	5 (6.8)	1 (0.7)	88 (7.9)	**<0.001**	24.4	
Phone hotline	20 (3.7)	4 (3.8)	5 (9.4)	15 (7.4)	3 (4.1)	8 (5.5)	55 (4.9)	0.210	6.9	
Consultation without parents present	173 (32.2)	11 (10.5)	13 (24.5)	50 (24.5)	33 (44.6)	12 (8.2)	292 (26.1)	**<0.001**‡	61.4	5
Joint transition clinics with the paediatric and adult services	65 (12.1)	2 (1.9)	4 (7.5)	15 (7.4)	6 (8.1)	8 (5.5)	100 (8.9)	**0.005**	16.6	
Transition lead	33 (6.1)	0 (0.0)	0 (0.0)	11 (5.4)	1 (1.4)	3 (2.1)	48 (4.3)	**0.005**	15.7	
Transition network	26 (4.8)	2 (1.9)	0 (0.0)	4 (2.0)	4 (5.4)	2 (1.4)	38 (3.4)	0.093	8.8	
Transition report	54 (10.1)	18 (17.1)	7 (13.2)	71 (34.8)	2 (2.7)	11 (7.5)	163 (14.6)	**<0.001**‡	90.7	5
Consultation letters or reports are sent to paediatric or adult colleagues involved in individual patients' care	115 (21.4)	22 (21.0)	17 (32.1)	73 (35.8)	38 (51.4)	11 (7.5)	276 (24.7)	**<0.001**‡	70.4	5
Communication addressed directly to the AYA	105 (19.6)	8 (7.6)	8 (15.1)	36 (17.6)	24 (32.4)	6 (4.1)	187 (16.7)	**<0.001**‡	39.4	5
Regular meetings involving paediatric and adult services in the field of allergy and pneumology to discuss AYA patients	56 (10.4)	0 (0.0)	0 (0.0)	28 (13.7)	9 (12.2)	4 (2.7)	97 (8.7)	**<0.001**	38.6	

Analysis for Greek and French languages was not performed due to low number of responses. AYA, adolescents and young adults; DF, degree of freedom; HCP, healthcare professionals; NA, not applicable. [a] Participants were allowed to select more than 1 answer. Statistically significant P values are in boldface text. Determined by using †Fisher exact test and ‡χ2 test. P value less than 0.05 was considered statistically significant. The difference between responses in different languages where similar to the difference in response between difference countries (Table S2).

Table S2. Comparison of transition practices in seven European countries with more than 50 responses

	Germany (n=68)	Italy (n=110)	Portugal (n=56)	Russia (n=175)	Romania (n=54)	Spain (n=170)	UK (n=124)	P value†	χ2 value	DF
**Transition is important for AYA with allergies/asthma, n (%)**								**<0.001**	61.6	
Strongly agree	31 (45.6)	68 (62.4)	17 (38.6)	113 (65.3)	21 (42.0)	82 (52.6)	79 (63.7)			
Agree	18 (26.5)	36 (33.0)	16 (36.4)	41 (23.7)	20 (40.0)	45 (28.8)	40 (32.3)			
Neither agree nor disagree	16 (23.5)	4 (3.7)	7 (15.9)	18 (10.4)	8 (16.0)	22 (14.1)	4 (3.2)			
Disagree	3 (4.4)	0 (0.0)	2 (4.5)	1 (0.6)	1 (2.0)	5 (3.2)	0 (0.0)			
Strongly disagree	0 (0.0)	1 (0.9)	2 (4.5)	0 (0.0)	0 (0.0)	2 (1.3)	1 (0.8)			
**Time for usual follow-up consultation with AYA, n (%)**								**<0.001**	296.1	
Up to 10 min	27 (39.7)	4 (3.6)	0 (0.0)	2 (1.1)	1 (1.9)	54 (31.8)	5 (4.0)			
Up to 20 min	32 (47.1)	44 (40.0)	24 (42.9)	40 (22.9)	25 (46.3)	93 (54.7)	44 (35.5)			
Up to 30 min	8 (11.8)	45 (40.9)	31 (55.4)	74 (42.3)	23 (42.6)	18 (10.6)	49 (39.5)			
Up to 45 min	1 (1.5)	10 (9.1)	0 (0.0)	46 (26.3)	4 (7.4)	1 (0.6)	19 (15.3)			
> 45 min	0 (0.0)	7 (6.4)	1 (1.8)	13 (7.4)	1 (1.9)	4 (2.4)	7 (5.6)			
**HCP's category based on patient's age, n (%)** [a]								**<0.001**	165.0	
Paediatric HCP	20 (29.4)	68 (61.8)	10 (17.9)	54 (30.9)	7 (13.0)	43 (25.3)	87 (70.2)			
Adult HCP	0 (0.0)	3 (2.7)	2 (3.6)	27 (15.4)	6 (11.1)	1 (0.6)	4 (3.2)			
All ages groups HCP	48 (70.6)	39 (35.5)	44 (78.6)	94 (53.7)	41 (75.9)	126 (74.1)	33 (26.6)			
**Age of AYA when you usually start the transition process, n (%)**								**<0.001**	149.7	
NA, my clinic does not have a transition process	26 (41.3)	39 (35.8)	33 (63.5)	79 (46.2)	32 (59.3)	71 (42.8)	29 (23.4)			
10-12 years	1 (1.6)	3 (2.8)	0 (0.0)	3 (1.8)	0 (0.0)	1 (0.6)	4 (3.2)			
12-14 years	3 (4.8)	15 (13.8)	1 (1.9)	13 (7.6)	0 (0.0)	16 (9.6)	36 (29.0)			
14-16 years	16 (25.4)	28 (25.7)	1 (1.9)	23 (13.5)	3 (5.6)	49 (29.5)	38 (30.6)			
16-18 years	17 (27.0)	24 (22.0)	16 (30.8)	53 (31.0)	14 (25.9)	27 (16.3)	15 (12.1)			
>18 years	0 (0.0)	0 (0.0)	1 (1.9)	0 (0.0)	5 (9.3)	2 (1.2)	0 (0.0)			
Other[b]	0 (0.0)	0 (0.0)	0 (0.0)	0 (0.0)	0 (0.0)	0 (0.0)	2 (1.6)			
**Age when AYA transferred to adult services n (%)**								**<0.001**	343.5	
NA, not transfer into adult services	10 (16.1)	15 (14.2)	7 (13.0)	31 (17.8)	6 (11.1)	20 (12.7)	16 (12.9)			
NA, see all ages	21 (33.9)	14 (13.2)	30 (55.6)	33 (19.0)	21 (38.9)	62 (39.2)	0 (0.0)			
NA, only adult patients	1 (1.6)	1 (0.9)	0 (0.0)	8 (4.6)	0 (0.0)	0 (0.0)	2 (1.6)			
By 14-15 years	0 (0.0)	1 (0.9)	0 (0.0)	0 (0.0)	0 (0.0)	21 (13.3)	1 (0.8)			
By 16 years	5 (8.1)	36 (34.0)	0 (0.0)	6 (3.4)	0 (0.0)	32 (20.3)	23 (18.5)			
By 17 years	3 (4.8)	3 (2.8)	0 (0.0)	0 (0.0)	0 (0.0)	6 (3.8)	19 (15.3)			
By 18 years	22 (35.5)	36 (34.0)	17 (31.5)	96 (55.2)	27 (50.0)	16 (10.1)	55 (44.4)			
By 19-20 years	0 (0.0)	0 (0.0)	0 (0.0)	0 (0.0)	0 (0.0)	1 (0.6)	2 (1.6)			
Other[c]	0 (0.0)	0 (0.0)	0 (0.0)	0 (0.0)	0 (0.0)	0 (0.0)	6 (4.8)			
**Evaluation of AYA in order to be sent to adult services, n (%)** [d]										
Patients transferred at a specific age	21 (30.9)	54 (49.1)	8 (14.3)	95 (54.3)	13 (24.1)	69 (40.6)	45 (36.3)	**<0.001**‡	42.8	6
Parental consent	10 (14.7)	14 (12.7)	3 (5.4)	5 (2.9)	4 (7.4)	11 (6.5)	28 (22.6)	**<0.001**‡	38.7	6

	Germany (n=68)	Italy (n=110)	Portugal (n=56)	Russia (n=175)	Romania (n=54)	Spain (n=170)	UK (n=124)	P value[†]	χ2 value	DF
Patient consent	15 (22.1)	13 (11.8)	5 (8.9)	8 (4.6)	3 (5.6)	8 (4.7)	39 (31.5)	**<0.001**[‡]	71.2	6
Checklist of questions/knowledge that AYA completes	2 (2.9)	5 (4.5)	2 (3.6)	4 (2.3)	1 (1.9)	4 (2.4)	18 (14.5)	**<0.001**	23.2	
Completion of adolescent transition tool	2 (2.9)	2 (1.8)	1 (1.8)	1 (0.6)	0 (0.0)	4 (2.4)	30 (24.2)	**<0.001**	70.9	
NA, we see all ages	28 (41.2)	22 (20.0)	37 (66.1)	48 (27.4)	35 (64.8)	76 (44.7)	4 (3.2)	**<0.001**[‡]	125.5	6
NA, my clinic does not transfer patients to adult services	5 (7.4)	16 (14.5)	6 (10.7)	31 (17.7)	6 (11.1)	15 (8.8)	20 (16.1)	0.140[‡]	9.7	6
**Resources and other clinic elements to support AYA with allergies/asthma in your service, n (%)** [d]										
We have no specific resources for AYA	33 (48.5)	79 (71.8)	28 (50.0)	99 (56.6)	35 (64.8)	137 (80.6)	44 (35.5)	**<0.001**[‡]	74.9	6
Transition guideline for HCP	3 (4.4)	11 (10.0)	10 (17.9)	33 (18.9)	7 (13.0)	5 (2.9)	48 (38.7)	**<0.001**[‡]	82.6	6
Transition readiness assessment tool	2 (2.9)	2 (1.8)	0 (0.0)	4 (2.3)	3 (5.6)	2 (1.2)	39 (31.5)	**<0.001**	96.8	
Workshops	4 (5.9)	7 (6.4)	2 (3.6)	19 (10.9)	4 (7.4)	7 (4.1)	9 (7.3)	0.319	6.9	
Peer learning/peer support for AYA	6 (8.8)	7 (6.4)	3 (5.4)	11 (6.3)	2 (3.7)	4 (2.4)	7 (5.6)	0.404	6.0	
Webinars	5 (7.4)	4 (3.6)	1 (1.8)	4 (2.3)	3 (5.6)	0 (0.0)	1 (0.8)	**0.005**	15.3	
e-Learning materials	5 (7.4)	3 (2.7)	7 (12.5)	18 (10.3)	8 (14.8)	4 (2.4)	4 (3.2)	**<0.001**	22.7	
Phone hotline	3 (4.4)	4 (3.6)	6 (10.7)	13 (7.4)	0 (0.0)	9 (5.3)	5 (4.0)	0.174	8.6	
Consultation without parents present	27 (39.7)	12 (10.9)	15 (26.8)	43 (24.6)	8 (14.8)	16 (9.4)	59 (47.6)	**<0.001**[‡]	80.4	
Joint transition clinics with the paediatric and adult services	5 (7.4)	2 (1.8)	4 (7.1)	13 (7.4)	1 (1.9)	12 (7.1)	33 (26.6)	**<0.001**	46.3	
Transition lead	0 (0.0)	0 (0.0)	1 (1.8)	8 (4.6)	0 (0.0)	4 (2.4)	28 (22.6)	**<0.001**	63.3	
Transition network	4 (5.9)	2 (1.8)	0 (0.0)	3 (1.7)	0 (0.0)	2 (1.2)	6 (4.8)	0.110	8.9	
Transition report	2 (2.9)	18 (16.4)	7 (12.5)	60 (34.3)	1 (1.9)	13 (7.6)	13 (10.5)	**<0.001**[‡]	75.5	6
Consultation letters are sent to paediatric/adult colleagues involved with the case	33 (48.5)	23 (20.9)	17 (30.4)	61 (34.9)	10 (18.5)	10 (5.9)	42 (33.9)	**<0.001**[‡]	68.6	6
Communication addressed directly to the AYA	20 (29.4)	9 (8.2)	8 (14.3)	32 (18.3)	6 (11.1)	7 (4.1)	30 (24.2)	**<0.001**[‡]	41.7	6
Regular meetings involving paediatric and adult services	9 (13.2)	0 (0.0)	0 (0.0)	24 (13.7)	5 (9.3)	7 (4.1)	13 (10.5)	**<0.001**	35.3	
**Feedback system between paediatric and adult service, n (%)** [d]										
No system of feedback in place	27 (39.7)	76 (69.1)	12 (21.4)	93 (53.1)	19 (35.2)	59 (34.7)	77 (62.1)	**<0.001**[‡]	64.6	6
Regular meetings to discuss patients	6 (8.8)	2 (1.8)	2 (3.6)	30 (17.1)	1 (1.9)	20 (11.8)	12 (9.7)	**<0.001**[‡]	26.1	6
The consultation letter from the first visit to the adult clinic is sent back to the referring paediatrician	11 (16.2)	6 (5.5)	1 (1.8)	10 (5.7)	5 (9.3)	7 (4.1)	33 (26.6)	**<0.001**[‡]	59.6	6
Not applicable, we see all ages	30 (44.1)	26 (23.6)	41 (73.2)	54 (30.9)	35 (64.8)	87 (51.2)	4 (3.2)	**<0.001**[‡]	138.1	6
**Specific training in the care of AYA with allergies/ asthma, n (%)** [d]										
Dedicated training course	2 (2.9)	12 (10.9)	15 (26.8)	23 (13.1)	6 (11.1)	15 (8.8)	16 (12.9)	**0.004**[‡]	19.3	6
Online training course	0 (0.0)	2 (1.8)	0 (0.0)	10 (5.7)	5 (9.3)	8 (4.7)	10 (8.1)	**0.016**	14.5	
Supervision within clinic	7 (10.3)	7 (6.4)	16 (28.6)	24 (13.7)	6 (11.1)	21 (12.4)	18 (14.5)	**0.009**[‡]	17.2	6
No specific training	61 (89.7)	92 (83.6)	29 (51.8)	133(76.0)	41 (75.9)	132 (77.6)	85 (68.5)	**<0.001**[‡]	32.2	6

AYA, adolescents and young adults; DF, degree of freedom; HCP, healthcare professionals; NA, not applicable; UK, United Kingdom. [a]Paediatric HCP looking after 0-18 years old patients; adult HCP looking after ≥18 years old patients. [b]Depending on the developmental stage, food safety skills and reaction recognition/management. [c]After secondary school; depending on the wishes of the AYA, family, agreement with adult service. [d]Participants were allowed to select more than 1 answer.

Statistically significant P values are in boldface text. [†]Determined by using †Fisher exact test and [‡]χ^2 test. P value less than 0.05 was considered statistically significant.

Figure S1. Age at a start of transition process based on clinic type in seven countries with more than 50 responses

a) All ages groups HCP

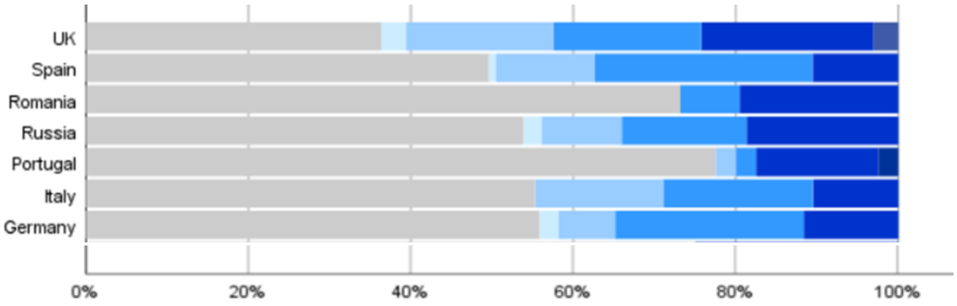

b) Paediatric HCP

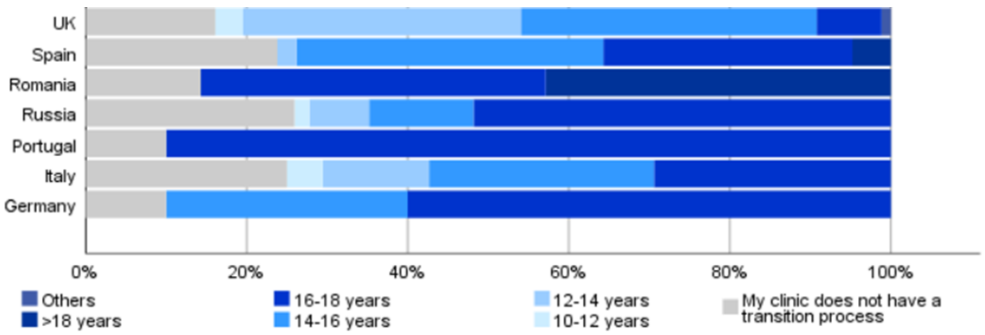

HCP, healthcare professionals; UK, United Kingdom. Paediatric HCP looking after 0-18 years old patients. All ages groups HCP looking after all age groups of patients. There was insufficient number of adult HCP for comparison. There were significant differences between countries (p<0.001) with significant interactions between clinic type and countries (multinomial logistic regression analysis, no shown).

Table S3. Criteria for transfer of adolescents and young adults with allergy and asthma to adult medical services in Europe

	n (%)
Asthma	119 (10.1)
Difficult, severe or poorly controlled asthma	356 (30.2)
Severe or uncontrolled atopic dermatitis	255 (21.6)
Any food allergy	92 (7.8)
Multiple food allergies	181 (15.4)
Food allergy who also have AAI	191 (16.2)
Anaphylaxis	276 (23.4)
Hymenoptera venom allergy	197 (16.7)
Multiple food allergies and asthma	229 (19.4)
Multiple food allergies, asthma and AAI	226 (22.6)
AIT for respiratory allergy	293 (23.9)
AIT for hymenoptera venom allergy	284 (24.1)
AIT for food allergy	133 (11.3)
Biological therapy: asthma/CSU/ angioedema	319 (27.1)
My clinic refers all patients to adult services	156 (13.2)
My clinic refers no patients to adult services	167 (14.2)
We see all ages	384 (32.6)

AYA, adolescent or young adult; AAI, adrenaline auto-injector; AIT, allergen immunotherapy; CSU, chronic spontaneous urticaria. 1179 participants contributed to the statistical analysis. Participants were allowed to select more than 1 answer.

Figure S2. Challenges for HCPs based on clinic type in Europe. Do you routinely ask about the following areas?

a) All ages groups HCP

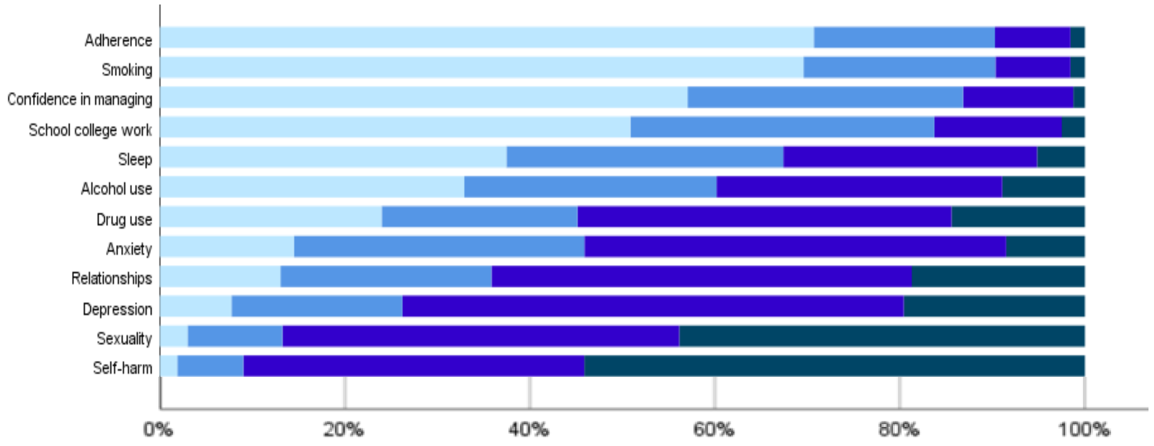

b) Adult HCP

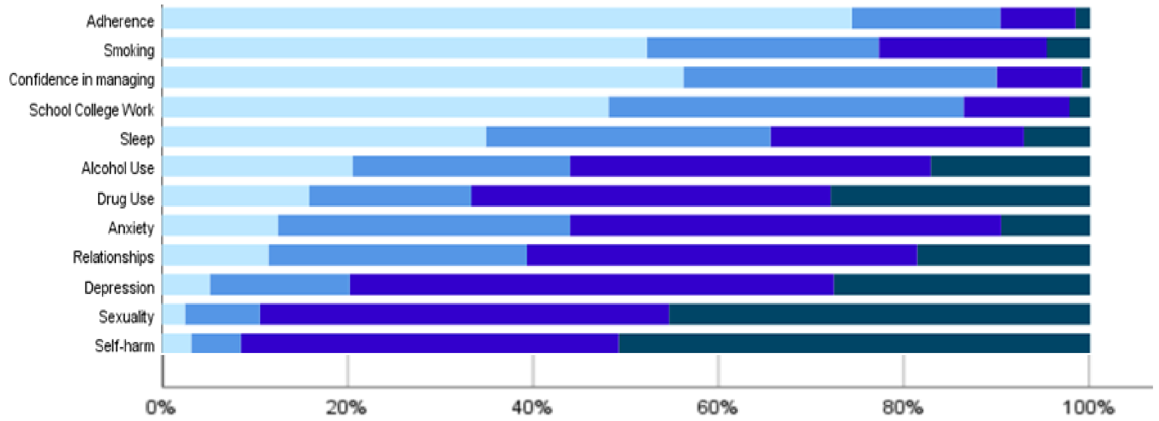

c) Paediatric HCP

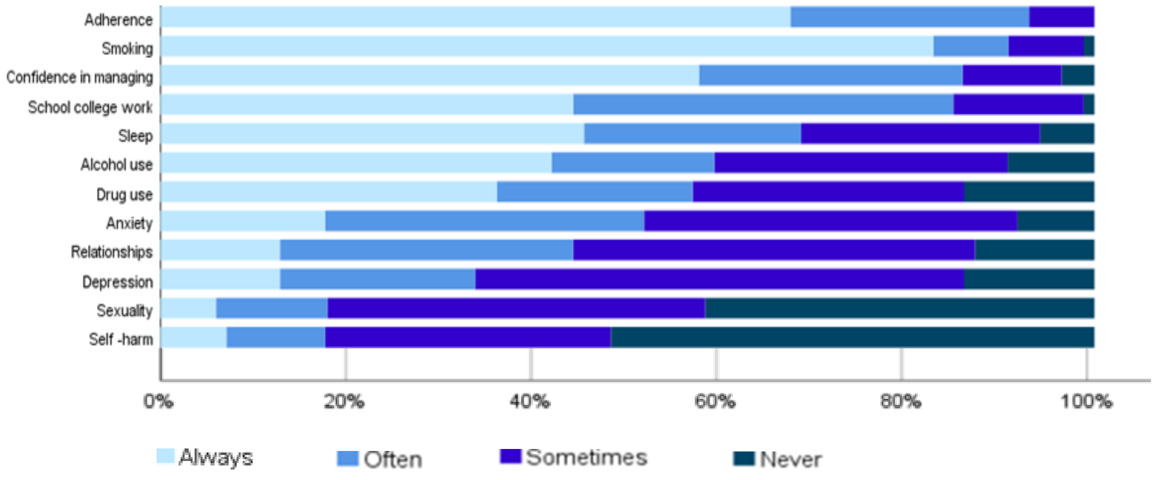

HCP, healthcare professionals. Paediatric HCP (n=449) looking after 0-18 years old patients; adult HCP (n=88) looking after ≥18 years old patients; all ages groups HCP (n=642). 1179 participants contributed to the statistical analysis.

Figure S3. Specific training in adolescents and young adults transition process

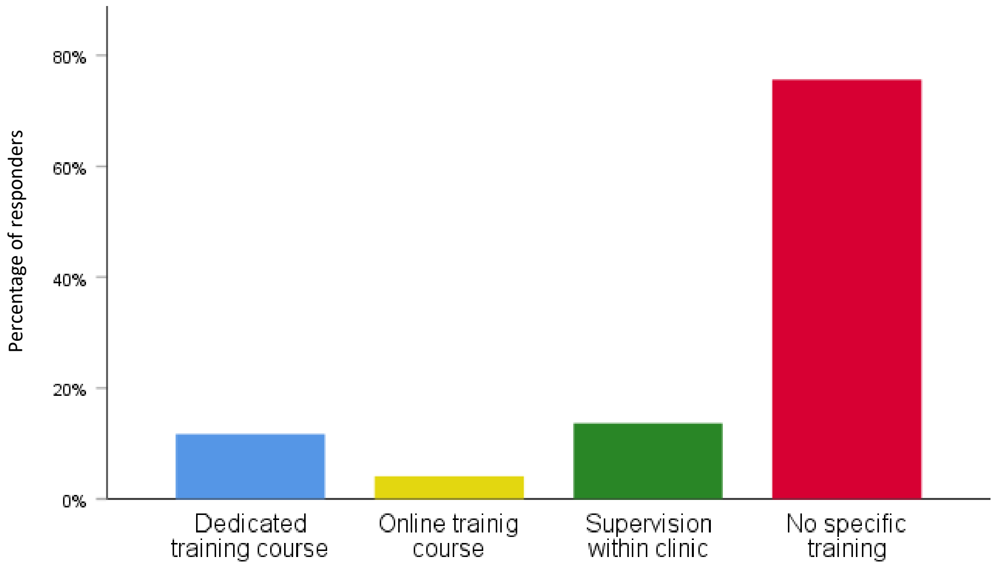

1179 participants contributed to the analysis. Participants were allowed to select more than 1 answer.

Figure S4. Have you had any specific training in the care of adolescents and young adults such as dedicated training course, online training course or supervision within clinic? Results are shown for 7 countries with more than 50 responses and based on type of clinic

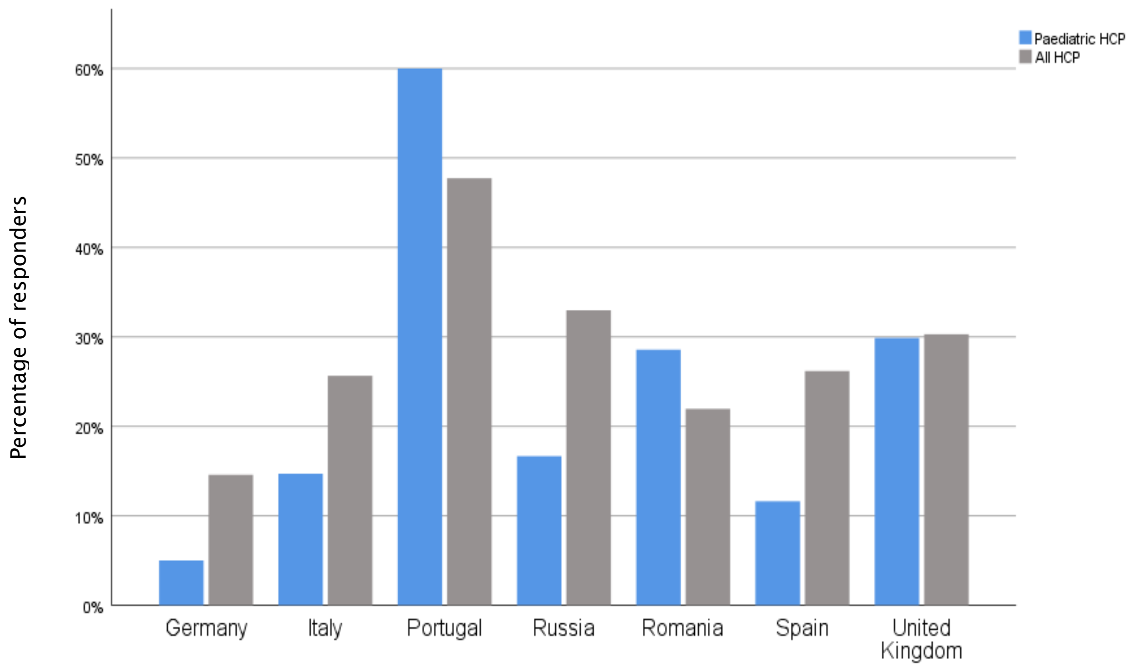

HCP, healthcare professionals. Paediatric HCP looking after 0-18 years old patients. All HCP looking after all age groups of patients. There was insufficient number of adult HCP for comparison. 714 participants contributed to the analysis.

Table S4. Specific training in management of adolescents and young adults with allergy and asthma based on type of clinic in 7 countries

	Coefficient	95% confidence interval	p-value
Paediatric HCP	Reference		
All age groups HCP	0.033	0.004, 0.062	0.027
Germany	Reference		
Italy	0.124	0.014, 0.233	0.027
Portugal	0.223	0.096, 0.349	0.001
Russia	0.144	0.042, 0.246	0.006
Romania	0.128	-0.004, 0.259	0.057
Spain	0.092	-0.007, 0.192	0.070
United Kingdom	0.190	0.082, 0.299	0.001

Numbers show coefficients for regression analysis for specific training in management of adolescents and young adults with allergy and asthma; a positive coefficient indicates that that group of respondents were more likely to have received training. A total of 714 participants contributed to the analysis. There were insufficient numbers of adult healthcare practitioners (HCP) for inclusion. There were no significant interactions between clinic type and country.

Figure S5. Agreement of healthcare professionals with the statement: "transition is important for adolescents and young adults with allergies or asthma''

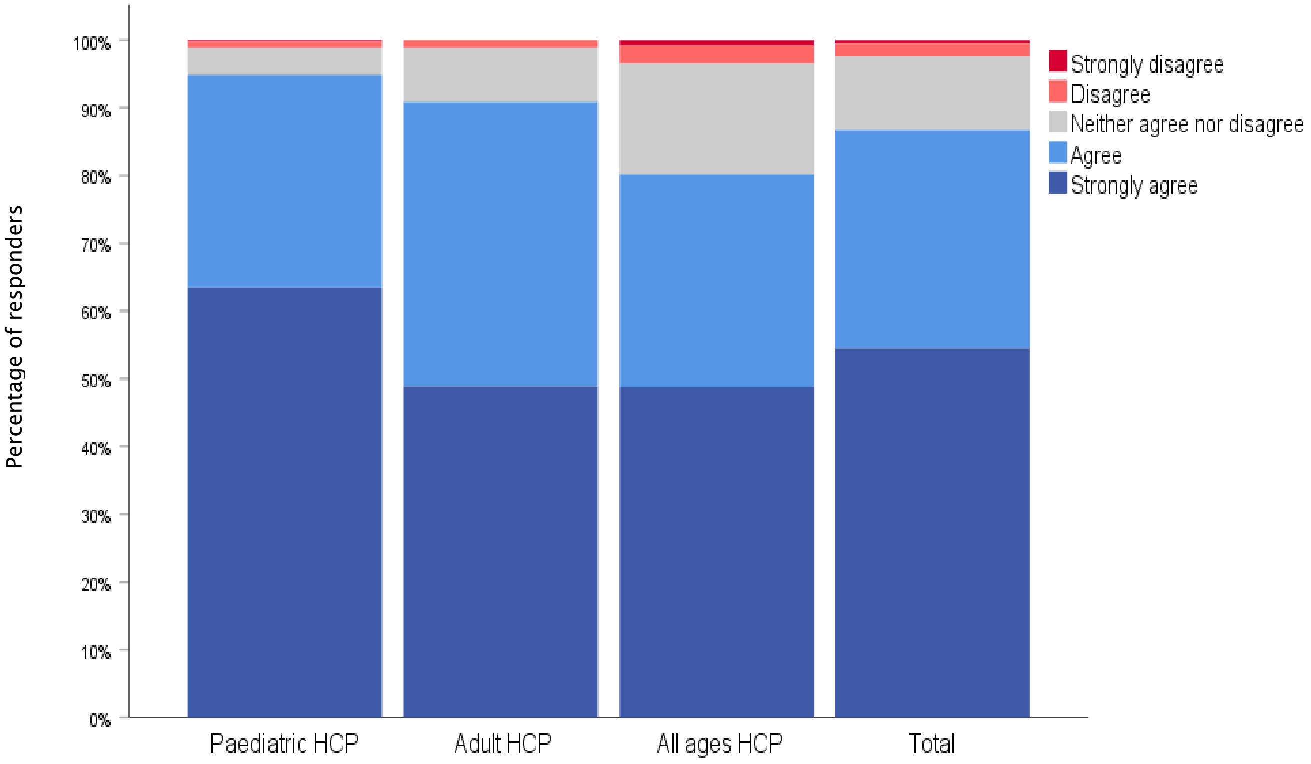

HCP, healthcare professionals. Paediatric HCP (n=449) looking after 0-18 years old patients; adult HCP (n=88) looking after ≥18 years old patients; all ages HCP (n=642) looking after all age groups of patients. 1179 participants contributed to the statistical analysis.

Figure S6. Agreement of healthcare professionals with the statement: "transition is important for adolescents and young adults with allergies or asthma'' based on clinic type in countries with more than 50 responses

a) All age groups HCP

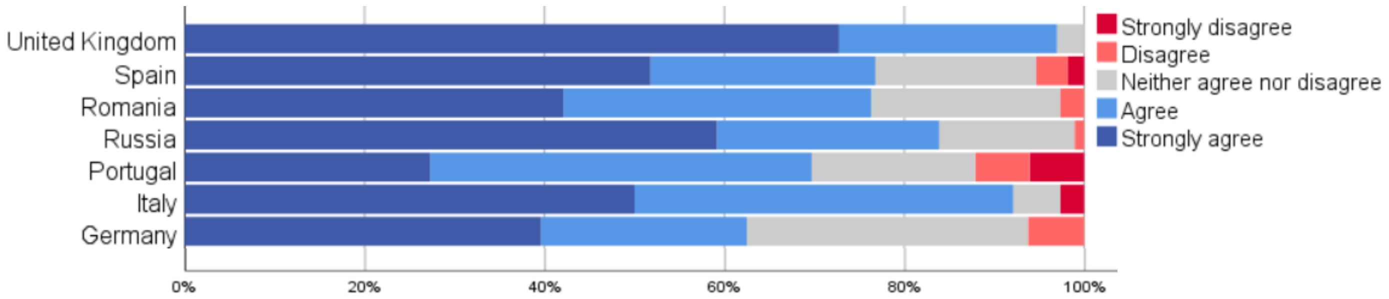

b) Paediatric HCP

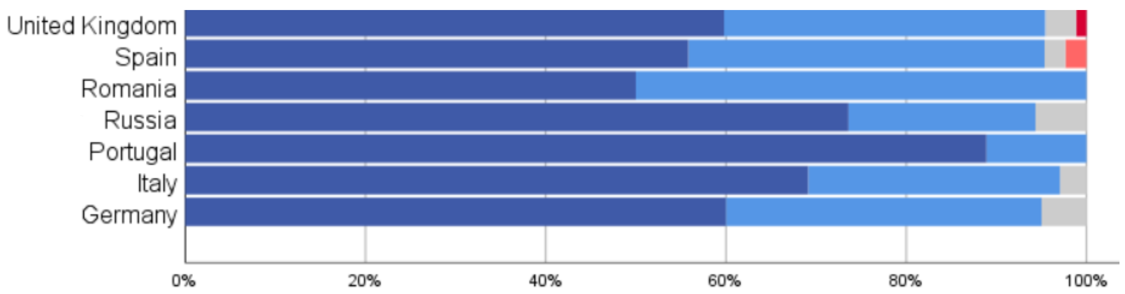

HCP, healthcare professionals. Paediatric HCP looking after 0-18 years old patients; All age groups HCP looking after all age groups of patients. There was insufficient number of adult HCP for comparison. 757 participants contributed to the analysis.

Table S5. Agreement of healthcare professionals with the statement: "transition is important for adolescents and young adults with allergies or asthma'' based on clinic type in countries with more than 50 responses

	Coefficient	95% confidence interval	p-value
Paediatric HCP	Reference		
All ages groups HCP	0.084	-0.074, 0.242	0.297
Germany	Reference		
Italy	0.112	-0.282, 0.505	0.577
Portugal	0.339	-0.282, 0.960	0.284
Russia	0.129	0.277, 0.535	0.532
Romania	-0.050	-0.770, 0.670	0.892
Spain	-0.062	-0.480, 0.357	0.773
United Kingdom	-0.213	-0.405, 0.362	0.913
All ages*Germany	-0.760	-1.279, -0.241	0.004
All ages*Italy	-0.462	-0.907, -0.016	0.042
All ages*Portugal	-1.269	-1.931, -0.607	<0.001
All ages*Russia	-0.428	-0.842, -0.015	0.042
All ages*Romania	-0.510	-1.260, 0.240	0.182
All ages*Spain	-0.442	-0.863, -0.021	0.039
All ages*United Kingdom	omitted		

Numbers show coefficients for regression analysis with agreement with the statement as the outcome (strongly disagree: -2; disagree: -1; neither agree nor disagree: 0; agree: 1; strongly agree: 2). Positive coefficient indicate that that group of respondents were more likely to be positive about the statement. A total of 757 participants contributed to the analysis. There were insufficient numbers of adult healthcare professionals (HCP) for inclusion. Bottom band are the interaction terms.

Figure S7. Does the department of health in your country make transition process a high priority?

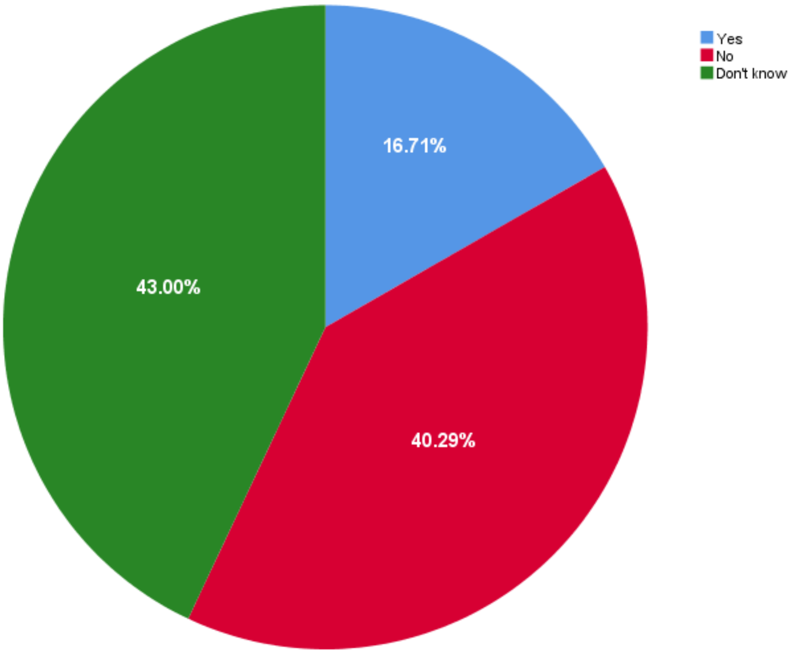

1179 participants contributed to the statistical analysis.
